# Tissue Harvester with Functional Valve (THFV): Shidham's device for reproducibly higher specimen yield by fine needle aspiration biopsy with easy to perform steps

**DOI:** 10.1186/1472-6890-7-2

**Published:** 2007-03-07

**Authors:** Vinod B Shidham, Ashwini W Pandit, R Nagarjun Rao, Zainab Basir, Anjani Shidham

**Affiliations:** 1 Dept of Pathology, Medical College of Wisconsin, Milwaukee, WI, USA; 2 Bioinnovation LLC, Elm Grove, WI, USA

## Abstract

**Background:**

Fine needle aspiration biopsy (FNAB) cytology has been a highly effective methodology for tissue diagnosis and for various ancillary studies including molecular tests. In addition to other benefits, FNAB predominantly retrieves the diagnostic loosely cohesive cells in the lesion as compared to the adjacent supporting stroma with relatively higher cohesiveness. However, FNAB procedure performed with currently available resources is highly skill dependent with inter-performer variability, which compromises its full potential as a diagnostic tool. In this study we report a device overcoming these limitations.

**Methods:**

'Tissue Harvester with Functional Valve' (THFV) was evaluated as part of a phase 1 National Institute of Health (NIH) research grant under Small Business Technology Transfer (STTR) Program. Working prototypes of the device were prepared. Each of the four cytopathologists with previous cytopathology fellowship training and experience in performing FNAB evaluated 5 THFV and 5 hypodermic needles resulting in 40 specimens (20 with THFV, 20 with hypodermic needles). A piece of fresh cattle liver stuffed in latex glove was used as the specimen. Based on these results a finished design was finalized.

**Results:**

The smears and cell blocks prepared from the specimens obtained by THFV were superior in terms of cellularity to specimens obtained with hypodermic needles. The tissuecrit of specimens obtained with THFV ranged from 70 to 100 μl (mean 87, SD 10), compared to 17 to 30 μl (mean 24, SD 4) with conventional hypodermic needles (p < .0001, Student t-test). The technical ease [on a scale of 1 (easy) to 5 (difficult)] with THFV ranged from 1 to 2 as compared to 2 to 3 with hypodermic needles.

**Conclusion:**

The specimen yield with the new THFV was significantly higher when compared to hypodermic needles. Also, the FNAB procedure with THFV was relatively easier in comparison with hypodermic needles. The final version of Shidham's THFV device would improve the FNAB specimen yield by eliminating the skill factor. The increased specimen yield by this device would also facilitate wider application of FNAB specimens for various ancillary tests, including molecular tests.

## Background

**Figure 1:**
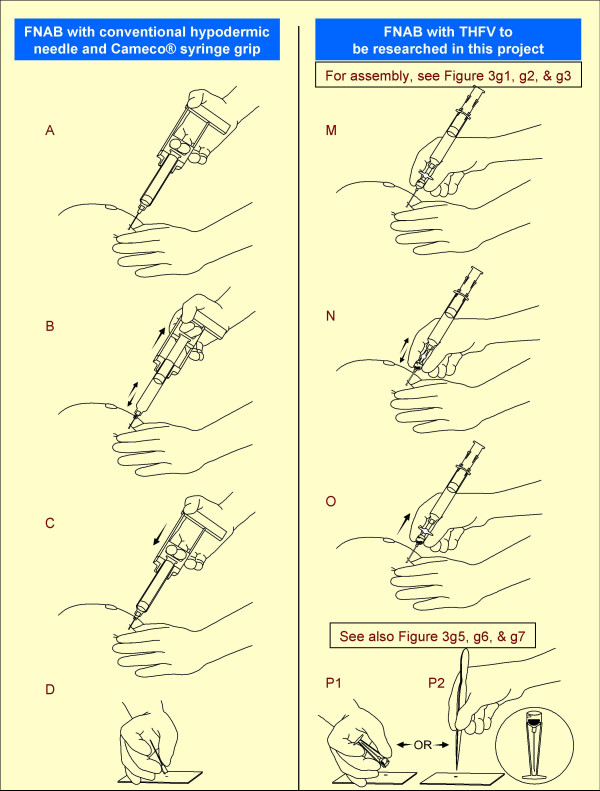
Comparison of FNAB procedures by hypodermic needle and THFV.

Fine Needle Aspiration Biopsy (FNAB) is a safe, rapid, economical, and minimally invasive non-surgical technique for tissue diagnosis of various tumors and lesions [1-15]. It is widely performed around the world including in more than six thousand hospitals in the United States [16,17]. FNAB is a procedure in which a fine gauge needle (ranging from 18G to 25G, preferably 23 to 25G) is inserted into a lesion and moved in multiple directions for wider sampling, usually under vacuum, to retrieve cells and microfragments of tissue (Figure 1 A through D).

Due to the non-availability of special FNAB needles, conventional hypodermic needles or their minor modified versions have to be used to perform the procedure. The limitations of conventional needles include inherent deficiencies in their design, and the requirement of greater technical skill, which leads to a higher frequency of unsatisfactory results, and a relatively low specimen yield.

One of the major limiting factors of a conventional hypodermic needle is the narrow diameter and low volume of the hub (Figure 1D). This restricts the quantity of specimen material that can accumulate in the hub at the end of the FNAB procedure without entering the syringe. If the scant material entering the syringe spreads along the syringe barrel wall, it cannot be retrieved properly for preparation of direct smears for cytopathologic evaluation. Accordingly, it is important for the performer to be aware of the material entering the syringe and stop the procedure at that time. These factors contribute to qualitatively and quantitatively poor diagnostic material with a higher proportion of unsatisfactory results.

Special syringe grips (Figure 1A-C) further increase the complexity of the procedure [18]. Thus, due to the lack of a commercially available suitable device for performing FNAB, this widely used valuable technique suffers significant shortcomings with lower reproducibility because of variable skills and success rates.

Depending on initial cytopathologic evaluation, additional ancillary tests such as flow cytometry, molecular techniques, cytogenetics, microbiology cultures, fluorescent in-situ hybridization, electron microscopy, cell block for immunocytochemistry, or other tests may be indicated. With currently available methods, this requires that additional passes be performed to obtain more sample. This increases patient discomfort and leads to higher utilization of disposables.

**Figure 2:**
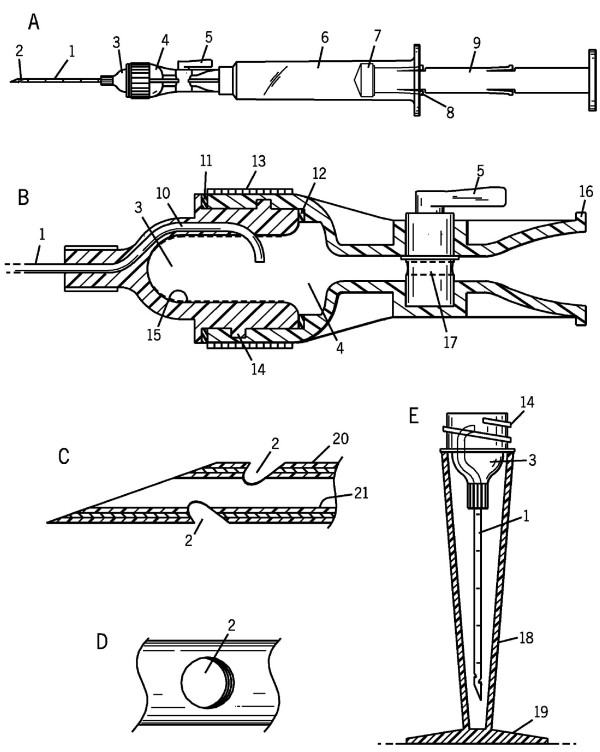
Initial design for planning three dimensional CAD.

The aim of this study was to prepare the device prototype and evaluate the efficiency of the initial design for specimen retrieval (Figure 2). The evaluation of efficiency included comparison with the traditional method. Based on the experience with the prototype, the final design for mass production was refined further at the end of the study.

## Methods

**Figure 3:**
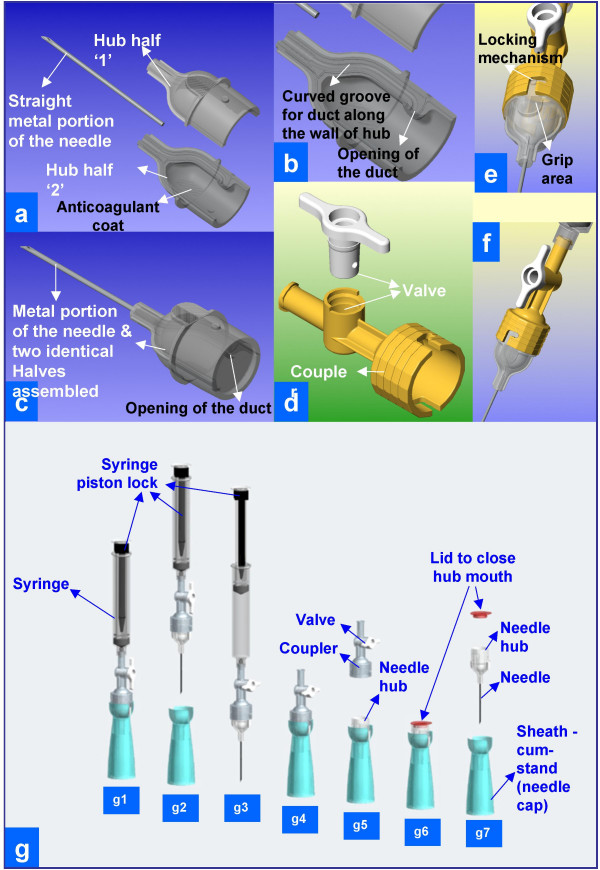
Three dimensional CAD as initial step towards preparation of SLA prototype.

**Figure 4:**
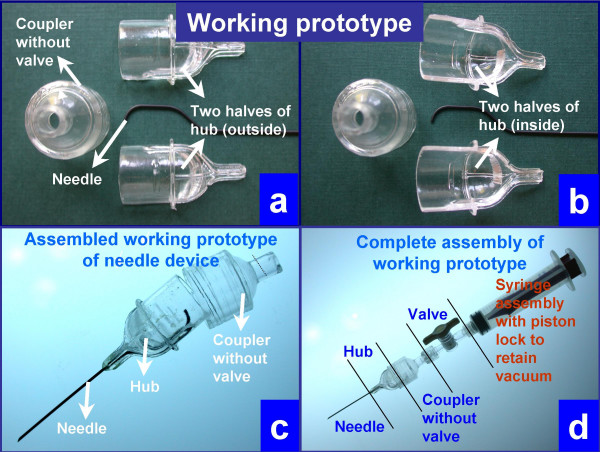
Working prototype to be evaluated in the study.

As part of a phase 1 NIH research grant under Small Business Technology Transfer (STTR) Program, the novel design of THFV by authors VS and AS was evaluated by cytopathology fellowship trained cytopathologists [19]. Rapid prototypes (Figures 3 and 4) of THFV device were prepared by Stereolithography (SLA) after critical evaluation of three dimensional computer assisted design (CAD) using SolidWorks^® ^software (SolidWorks Corporation, Concord, MA). Working prototypes of clear polyurethane were prepared using Silicone RTV molds set around SLA masters (Figure AF3 in Additional file #1). Each cytopathologist (VS,AP,RNR,ZB) evaluated 5 working models of THFV devices (Figure 4) and compared the results with 5 hypodermic needles by the same cytopathologists. This generated 20 FNABs by THFV devices and 20 by hypodermic needles. A piece of fresh cattle liver procured from the local slaughter house was stuffed in latex glove and were used as specimens. Cellularity of direct smears, Tissuecrit of the needle rinses, and technical ease of performing the procedure were compared.

### Evaluation of the specimen yield of the working models to demonstrate the feasibility of the invention

Various steps during the procedure with the conventional hypodermic needle and with the new needle device are shown in Figure 1. The procedure for performing the FNAB with the working model of Shidham's THFV is as follows:

(a) Assemble the needle hub, the coupler with tap like valve, and the syringe as shown in Figure 3g2 and 4d.

(b) Close the tap like valve (Figure 3g3).

(c) Create vacuum in the syringe barrel by pulling the syringe piston, which locks itself in the pulled position (Figure 3g3).

(d) Grip the needle device like a pencil at the wide mouth hub (Figure 1M-O).

(e) Insert the needle into the lesion to be sampled under routine aseptic precautions.

(f) Once the needle is in the lesion, open the tap like valve of the coupler to facilitate the suction effect of the vacuum in the syringe to reach up to the needle tip.

(g) Push the needle back and forth into the lesion in different directions for optimal sampling of the lesion till enough material accumulates in the transparent wide-mouth hub (Figure 1N).

(h) Close the tap like valve of the coupler to disconnect the vacuum in the syringe from the needle.

(i) Remove the needle from the lesion and park the needle into the cap cum stand-like cradle without holding the stand with hand to avoid needle prick injury. The needle fits into the cap-cum-stand (Figure 3g4).

(j) Disengage the wide mouth hub of the needle from the coupler by holding the grip area on the hub (Figures 3g5) with the needle in the cap cum stand.

(k) Transfer the representative sample by inverting the capped needle gently on the glass slide or by using a fine tip forceps to transfer the microfragments from wide mouth hub with 'pick and smear' method [20]. During this, the needle could be parked in upright position in the cap cum stand (Figure 1P1,P2) with wide base (Figures 1P2, 3g, and 5d).

**Figure 5:**
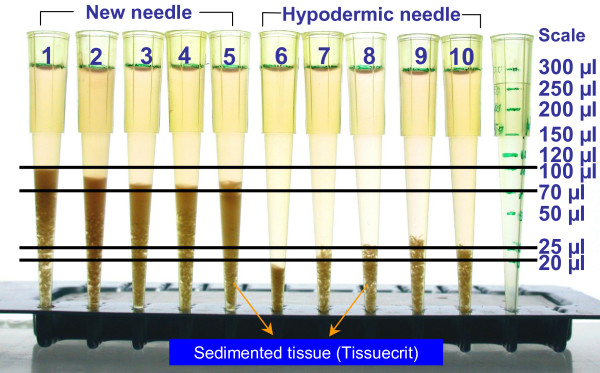
Comparison of Tissuecrits.

The remaining specimen in the wide mouth hub was rinsed with Cytorich for estimation of Tissuecrit, which is the volume of tissue microfragments. The needle hub was rinsed with a constant volume (300 μl) of CytoRich Red^® ^fixative (AutoCyte Inc, Elon College, NC) for cell block preparation after preparing direct cytology smears. The tissue micro-fragments were compacted as sediments under gravity for 60 minutes and the volume of the tissue fragments was noted (Figure 5).

Cellularity of the direct smears with the new needle was evaluated semi-quantitatively (hypocellular- very few scattered cells, mild- <20% area in the field covered by cells in the specimen, moderate- 20% to 50% area in the field covered by cells in the specimen, and marked- >50% area in the field covered by cells in the specimen). The technical ease of performing the procedure was also noted by each pathologist on a scale of 1 (easy) to 5 (difficult).

## Results

**Figure 6:**
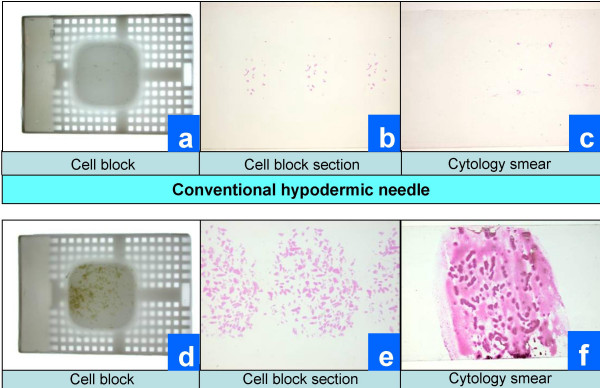
Gross comparison of cellularity in cell-block and cytology smears.

**Figure 7:**
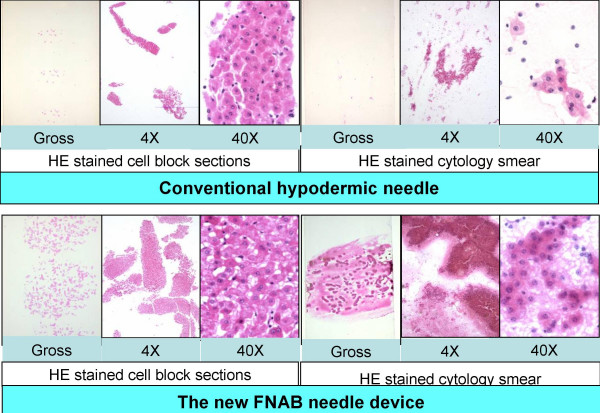
Microscopic comparison of cellularity in cell-block and cytology smears.

The Tissuecrit of specimens obtained with THFV ranged from 70 to 100 μl (mean 87, SD 10) as compared to 17 to 30 μl (mean 24, SD 4) with SN (p < .0001, Student t-test). Tissuecrit (Figure 5) showed a higher yield with the new needle device when compared to the FNAB results with conventional hypodermic needles and demonstrated the feasibility of the invention (Table 1). The smears (Figure 6 c&f; 7) and cell blocks (Figure 6 a,b & d,e; 7) prepared from the specimens obtained by THFV were uniformly hypercellular as compared to mostly hypocelluar with hypodermic needles. The technical ease with THFV ranged from 1 to 2 as compared to 2 to 3 with hypodermic needles.

**Table 1:** Comparison of results of the new THFV with those with conventional hypodermic needles.

Feature	New FNAB needle device (5 by each of 4 CP-Total 20)	Hypodermic needle (5 by each of 4 CP-Total 20)
**Cellularity of direct smears**	Moderate to marked (Figure 6c)	Mild to hypocellular (Figure 6f)
**Density of tissue fragments in cell block**	Moderate to marked (Figure 6a,b)	Mild to hypocellular (Figure 6d,e)
**Tissuecrit^¶ ^(Figure 5)**	70–100 μl (Mean 87, SD 10)*	17–30 μl (Mean 24, SD 4)*
**Technical ease§**	1–2	2–3

DS, direct smear, CP, cytopathologists, SD, standard deviation.

^¶^Volume of tissue microfragments procured with FNAB procedure and rinsed in CytoRich Red^® ^fixative (AutoCyte Inc, Elon College, NC) for cell block preparation after preparing direct cytology smears. The tissue microfragments were compacted as sediments under gravity for fixed duration (Figure 5).

*p value < .0001, Student t-test.

§Scale of 1 (easy) to 5 (difficult).

Perforations with the 45 degree bevel at the tip of the needle did not demonstrate any significant advantage with results compared to those achieved with needles without perforation in needle wall near the tip of the needle (Table AF1 #2 in Additional file #1). This feature was not considered a significant benefit in return for the cost and was removed in the final design.

## Discussion

The Shidham's THFV procured a significantly higher yield of FNAB specimen as compared to hypodermic needles. It demonstrated relatively superior technical ease as compared to hypodermic needles. The details of the new needle device are shown in Figure 2. A schematic representation of the procedures performed using THFV and a conventional hypodermic needle is shown in Figure 1. The mechanism of THFV is depicted in a brief animation (additional file 2).

The FNAB procedure is the method of choice for minimally invasive retrieval of tissue material from a patient for tissue diagnosis and other tests [21]. In contrast to FNAB, surgical operation requires special conditions such as an operating room, skilled surgical professionals, and supporting ancillary services. Furthermore, surgical biopsy also adds financial burden, pain, scarring, risk of complications, and loss of man hours with the invariable disruption of the personal schedule of the patient undergoing surgical biopsy. Many of the limitations of a surgical biopsy can be avoided by FNAB, which can be performed under general settings such as in the doctor's office. However, performing FNAB by conventional hypodermic needle with or without application of vacuum is associated with limitations of lower tissue yield which is usually insufficient for performing ancillary studies.

Many ancillary tests and evolving molecular techniques such as microarray and proteomics [13,22-24] require procurement of tissue specimens at various stages of management. It is critical to provide a reproducible procedure for such retrieval. THFV device would facilitate easily reproducible sampling with the added benefit of onsite cytologic evaluation to confirm the adequacy of specimen for the test to be performed. In addition to clinical applications, this device could also be used for precise harvesting of tissue for research from various specimens under aseptic conditions.

Although the modified version of the hypodermic needle with an elongated hub may allow slightly more specimen yield, it does not retain the entire specimen retrieved during the process. A significant portion of specimen in the needle and hub is lost back due to negative pressure and capillary action while withdrawing the needle from the lesion during the last step. Similar limitations apply to slightly modified hypodermic needles having a perforated tip.

**Figure 8:**
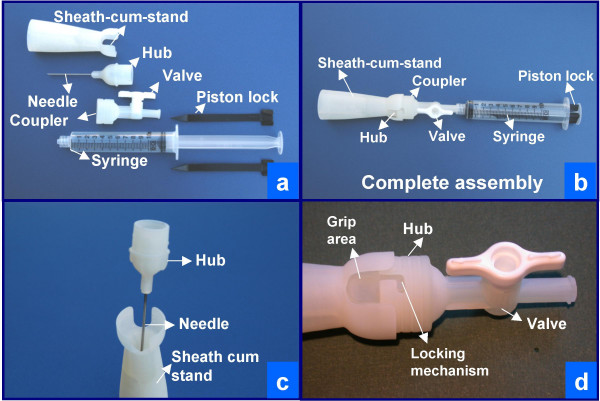
Final SLA model prepared after incorporating modifications based on the current study. Note that in final model coupler and valve are combined as one unit (a,b,d). Complete assembly is shown in b.

Once the specimen is retrieved, it is preferably examined immediately on site for adequacy evaluation with preliminary interpretation. A 'pick and spread' method allows smearing of selected material on the glass slide [20]. Due to the narrow mouth of the needle hub, hypodermic needles or their modified versions do not allow easy picking of micro-fragments in the specimen by fine tip forceps directly from the needle hub. The current device with wider hub allowed 'pick and spread' approach [20]. Based on the observations during this study [19] the design was finalized for the molds required for the mass production of "Shidhams' THFV device" (Figure 8).

## Conclusion

The new THFV device procured a significantly higher yield of FNAB specimen as compared to hypodermic needles. It demonstrated relatively superior technical ease as compared to hypodermic needles. This is a novel device for procurement of tissue for molecular tests and tissue diagnosis by various methods including cytopathology.

## List of abbreviations

3D, three dimensional; CAD, computer aided design; FNAB, Fine needle aspiration biopsy; RTV, Room Temperature Vulcanizing; SLA, Stereolithography; THFV, Tissue Harvester with Functional Valve.

## Competing interests

VS and AS, designed and invented the device for which US patent is filed by Medical College of Wisconsin. AS is CEO and Director of research at BioInnovations.

AP, RNR, and ZB do not have any competing interest in the device and its patent. They do not have any stake in BioInnovations. They are cytopathology and surgical pathology faculty at Medical College of Wisconsin

## Authors' contributions

**VS**, Designing and inventing the device with AS, conceptual organization of the study, analysis of the results, evaluating the device in comparison to traditionally used hypodermic needles by performing FNAB procedures on fresh liver specimens, and manuscript writing.

**AP**, **RNR**, and **ZB**, Evaluation of the device in comparison to traditionally used hypodermic needles by performing FNAB procedures on liver specimen, analysis of the results, and manuscript review.

**AS**, Designing and inventing the device with VS, conceptual organization of the study, and manuscript review.

All the authors read and approved the final manuscript.

## Pre-publication history

The pre-publication history for this paper can be accessed here:



## Supplementary Material

Additional File 1 Progression of prototype to working model. Details how the project progressed from prototype to working model. Click here for file

Additional File 2 Schematic animation of sampling process with THFV. Animation demonstrating the sampling of tissue fragments with THFV. Click here for file
