# Supplementary material for: Tissue Harvester with Functional Valve (THFV): Shidham's device for reproducibly higher specimen yield by fine needle aspiration biopsy with easy to perform steps
Source: BMC Clin Pathol. 2007 Mar 7;7:2. doi: 10.1186/1472-6890-7-2 (PMC1829396; doi:10.1186/1472-6890-7-2)
Supplement: Additional File 1 — Progression of prototype to working model. Details how the project progressed from prototype to working model. [file 1472-6890-7-2-S1.doc]

(Rev post 1^st^ PR 10-22-06)

**Additional file #2.**

**Progression from prototype to working model:**

**Tissue Harvester with Functional Valve (THFV): Shidham’s device for reproducibly higher specimen yield by fine needle aspiration biopsy with easy to perform steps.**

Vinod Shidham^1^, MD, FIAC, FRCPath ([vshidham@mcw.edu](mailto:vshidham@mcw.edu))

Ashwini Chavan^1^, MD, FIAC ([achavan@mcw.edu](mailto:achavan@mcw.edu))

R. Nagarjun Rao^1^, MD, FRCPath ([arao@mcw.edu](mailto:arao@mcw.edu))

Zainab Basir^1^, MD ([zbasir@mcw.edu](mailto:zbasir@mcw.edu))

Anjani Shidham^2^, BS ([ashidham@yahoo.com](mailto:ashidham@yahoo.com))

^1^Dept of Pathology, Medical College of Wisconsin, Milwaukee, WI; and

^2^Bioinnovation LLC, Elm Grove, WI.

**Corresponding author:**

**Vinod B. Shidham**, MD, FRCPath, FIAC
Professor

Director of Cytopath Fellowship Training Program and FNAB Service
Department of Pathology
Medical College of Wisconsin
9200 W Wisconsin Av
Milwaukee, WI 53226

Ph: (414) 805 8452
Fx: (414) 805 8444
Page: (414) 917 0732
[vshidham@mcw.edu](mailto:vshidham@mcw.edu)

Description, tables, and figures.

*Prototype* (Figure AF2) *of the new THFV device.*

A computer assisted three dimensional computer aided design (3D CAD) (Figure 2) was created by a vendor for Bioinnovation, LLC by using SolidWorks® software (SolidWorks Corporation, Concord, MA). The design was finalized after series of critical evaluations by VS and AS. The tolerances with SLA prototyping could not achieve the accuracies in dimension required for valve function. As a result, the coupler was prepared without the valve (Figure AF2a) to be used with the ready to use valve available on shelf (Figure 48d). To accommodate a tract for the curved portion of the needle traveling in the wall of the hub (Figure AF2d), the hub was prepared in two halves (Figure AF2b&d). Based on the finalized design, a rapid SLA prototype was prepared by the vendor (Figure AF2).

###### *Evaluation of the prototype.*

The SLA prototype (Figure AF2) was evaluated for various technical (such as incorporation of the curved needle portion in the hub) and ergonomic features (such as grip and ease of coupling of the hub with coupler) before proceeding with the next stage of preparing the ‘SLA master’ (Figure AF3) for the creation of RTV (Room Temperature Vulcanizing) silicone mold (Figure AF3) for production of multiple units of final working prototypes.

Teflon coated needles, curved according to the design, were procured by the vendor to be assembled with polyurethane plastic components for BioInnovation, LLC. The initial evaluation of this needle did not show any significant advantage with Teflon coating. So it was decided not to coat the needle with Teflon, and to use conventional polishing of surfaces similar to the ones used in the making of hypodermic needles.

*Production of many pieces of the working model.*

‘RTV silicone molds’ (Figure AF4) were prepared from an ‘SLA master’ (Figure AF3) for making many pieces of the ‘working prototypes’ (Figure 4) from polyurethane by the rapid prototype vendor. The rapid prototyping company incorporated the metal needle with it’s curved portion into the plastic component to generate the final working model (Figure 4c).

The basic model of the needle with its curved portion of the needle traveling along the side of the hub was modified slightly to evaluate if curving of the needle could be avoided in the final design (Table AF1). Curving of the metal portion of the needle is an expensive component and increases its final cost significantly. To economize the cost of the final FNAB needle device during commercialization, the curved portion of the needle (Figure AF2d) was replaced with the duct like channel along the wall of the hub (Figure 2b). This was done by comparison of the working prototype of basic design with curved needle (Figure AF2d; 4c) and other modified working models (Table AF1, Figure AF5).

The results with holes near the tip of needle (Table AF1 #2) were comparable to those without any holes in the needle wall near the tip of the needle (Table AF1 #1). With reference to this finding, the basic design of the needle without the hole in the needle wall near the needle tip was finalized. The devices with these needles were evaluated and compared with the conventional hypodermic needles. Other modifications tried similarly did not show any advantage. As high precision in dimensions could not be achieved with modified polyurethane models, the modification #6 and 7 (Table AF1, Figure AF5) could not be evaluated because of leakage problems.

The final basic design with curved needle along the hub wall (#1) was selected for this study (Table AF1). Based on this design 20 working models were prepared as rapid prototypes. The primary reason to finalize the curving of the needle was the inability to achieve precise tolerances with SLA models to prepare working prototype similar to modification 5 (Table AF1). It was decided that the model finalized for design of the mass production version would be the one with the hollow duct-like channel along the wall of the hub, instead of curved needle (Figure 2b).

**Comments:**

The working model prepared on the basis of the initial design was examined and improved by VS & AS (Figures 2, 8) with reference to the pencil grip of the hub, ease of dissembling the hub from coupler, locking of the hub with the coupler, incorporation of the hub with the valve, designing the flat lid to close the hub to facilitate the transportation of specimen to the lab if required, and the locking mechanism for the syringe piston to generate the vacuum during FNAB procedure.

The coating of the hub with anticoagulants to prevent the clotting of collected material during the procedure was part of the design, but was not evaluated as the aspirated material from fresh cattle liver did not clot. The benefits of anticoagulant coating during actual use of the device in patients would prevent clotting of specimen to allow some inherent delay in specimen processing.

Based on the comparisons (Table AF1), it was decided that the curved portion of the needle in the hub wall should be replaced by a duct-like channel continued along the wall of the hub (Table AF1, #5*), so that the metal portion of the needle could be straight to reduce the cost of the final product. This improvised relatively economical version of needle with a straight metal portion was finalized to prepare the molds for final production. However, the feasibility of the design was demonstrated with lumen of the straight needle continued as a duct-like channel in the hub wall with its terminal opening on the side wall of the hub away from the bottom of the hub (Figure 2).

A few modifications (Table AF1, Figure AF5) were evaluated on the basis of the pilot project and they did not show any advantage. Modification #6 and 7 could not be evaluated because of leakage problems due to the lack of exact precision in dimensions associated with these modified polyurethane models. However, these versions (#6 & 7) would have flexibility to adapt to any type of needles including traditional hypodermic needles and longer needles with coaxial components for deeper image guided FNABs. Although this modification #7 (Table AF1) could not be evaluated during this study, because of limitation of leakages associated with working prototype, the final version made by injection molding with precise tolerances should allow proper leak proof conditions for the results comparable to that with needle directly attached to the device.

Sheath-cum-stand was also designed and finalized to minimize chances of needle-prick injury. The optimum base diameter of 28 milimeters was deemed sufficient to maintain proper upright balance after parking the needle in it (Figure 2g4, g6).

**Table 2 (AF-1).**  Comparison between various modifications of the basic design of THFV (see Figure 9).

| S. No. | Modifications | Sketch | n | Smear cellularity | Remarks |
| --- | --- | --- | --- | --- | --- |
| 1* | Basic design with curved portion of the needle traveling along the hub wall (Figures 3d, AF2d) |  | 20 | Moderate-marked | Significantly larger volume of material accumulated at the bottom of needle hub. |
| 2 | Same as #1 with holes in the needle wall near the tip |  | 2 | Moderate-marked | Comparable to #1 |
| 3 | Straight needle ending at the bottom of wide mouth hub, similar to conventional hypodermic needles |  | 2 | Mild-moderate | The specimen ejects towards the end of the coupler attached to the valve. |
| 4 | Straight needle ending away from the bottom of wide mouth hub in its cavity |  | 2 | Mild-moderate | Same as #3 |
| 5^¶^ | Straight needle joining and continuing as the duct-like channel traveling along the hub wall and opening inside the hub away from its bottom (Figure 1b) |  | 2 | Moderate-marked | Comparable to #1 |
| 6 | Wide mouth hub without needle, but a spout to fit a conventional hypodermic needle. The spout opens at the bottom of the hub. |  | 1 | Could not be evaluated. | Due to low tolerances in the dimensions of modified polyurethane prototypes, there were leakage problems |
| 7 | Wide mouth hub without needle, but a spout to fit a conventional hypodermic needle similar to #6. The lumen of the spout connects to the duct-like channel traveling along the hub wall to open in the hub away from its bottom. |  | 1 | Could not be evaluated. | Same as #6. |

n= Numbers evaluated;

*The version# 1 was evaluated during phase I;

¶The version# 5 is selected as the structural principle, finalized for Phase II evaluation.

Figures (AF1 through AF5) for the additional file-

Finalization of the CAD design for the prototype by the rapid prototyping vendor.

Prepare a prototype of new needle device.

*Initial gross evaluation* of prototype for design features-

**a.** General ergonomics of the design

**b.** Air-tightness of junction between hub and coupler.

**c.** Closing / opening mechanism of the tap like valve.

**d.** Ease of tissue microfragment retrieval from the wide mouth hub by the fine tip forceps.

Dimensional, configurational, and other indicated *modifications* to finalize the prototype.

Prepare polyurethane *mold* for working model.

20 *working models*

**Conventional hypodermic needles**

*Performing FNAB* on fresh beef liver in a laboratory setting.

*Estimation* of tissue yield.

*Quantitative estimation* of *Tissuecrit*

(Figure 10)

*Statistical analysis* by ANOVA with Bonferroni method for multiple *comparisons*.

*Demonstration* of statistically significant *higher tissue yield*

**Feasibility demonstrated**

*Semiquantitative estimation* of cellularity of smears

(Figure 11 and 12).

*Semiquantitative estimation* of cell block sections

(Figure 11 and 12).

**Figure AF1**. Flow chart showing the research plan at a glance.


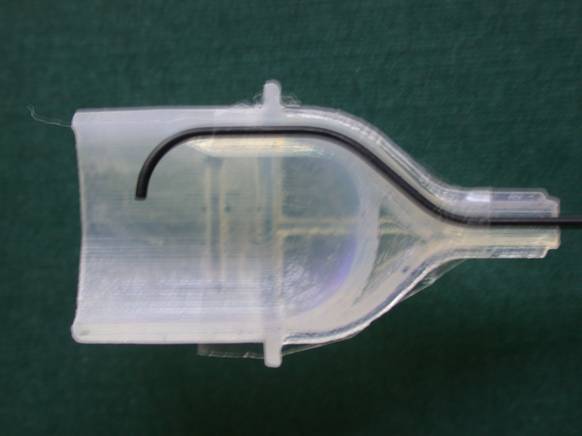


**Curved**

**portion**

**of needle**

**d**


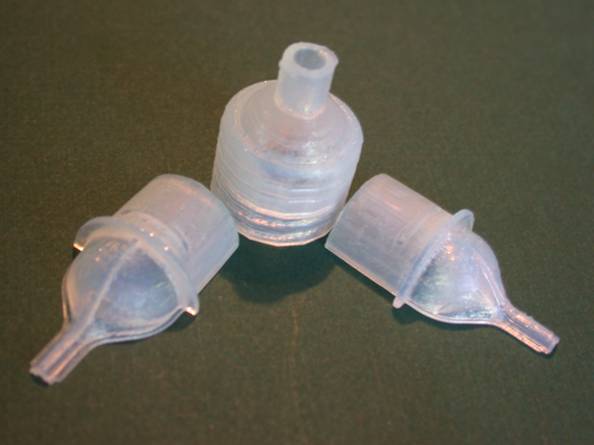


**Coupler**

**Two halves**

**of hub**

**b**


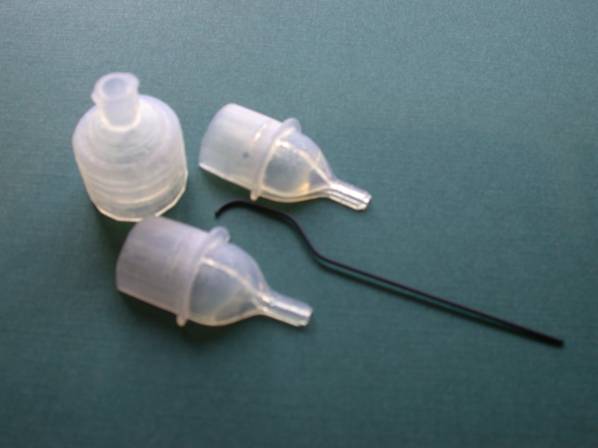


**Straight**

**portion**

**of needle**

**c**


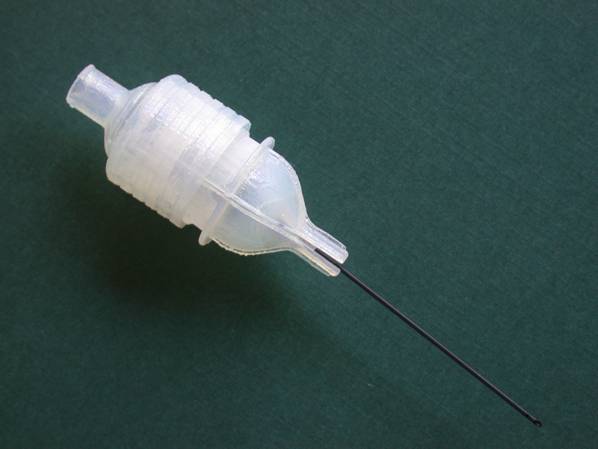


**Coupler**

**Hub**

**a**

**Figure AF2**. SLA Prototype. Because of technical limitations of achieving fine tolerances in dimensions with SLA prototypes, the coupler is without valve (a,b,c). On shelf valves (Figure 8d) available in the market were selected to be used.

**Hub half ‘1’**

**(inside view)**


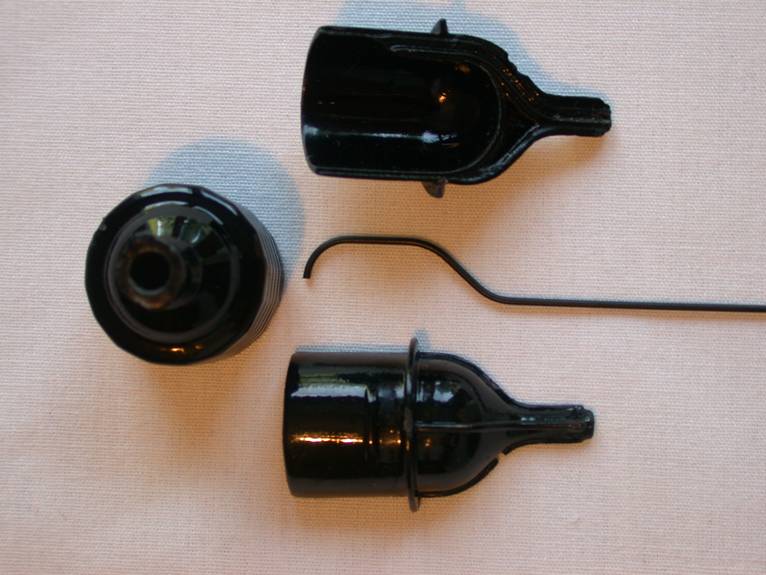


**Coupler**

**Needle**

**Hub half ‘1’**

**(inside view)**

**Hub half ‘2’**

**(outside view)**

**Figure AF3**. SLA master as physical form of CAD


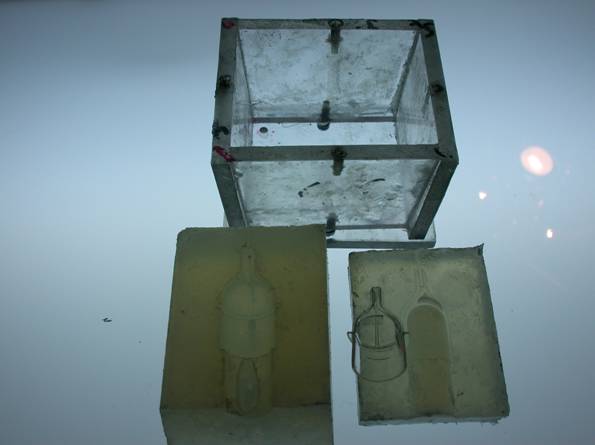

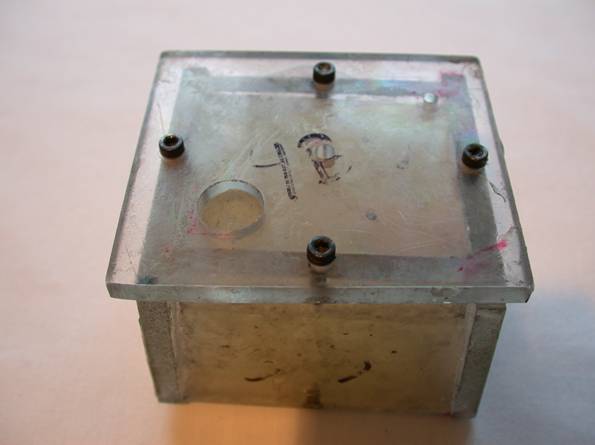

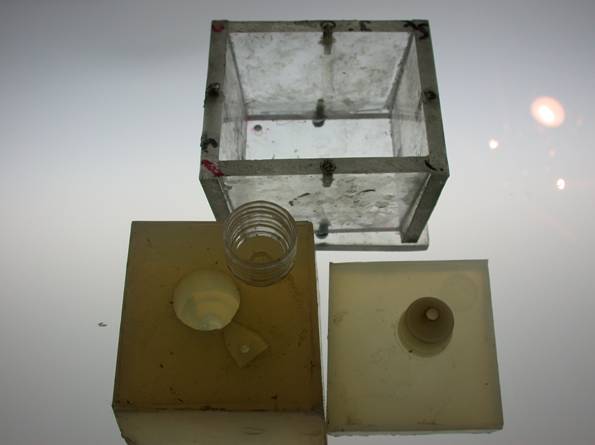


**RTV molds**


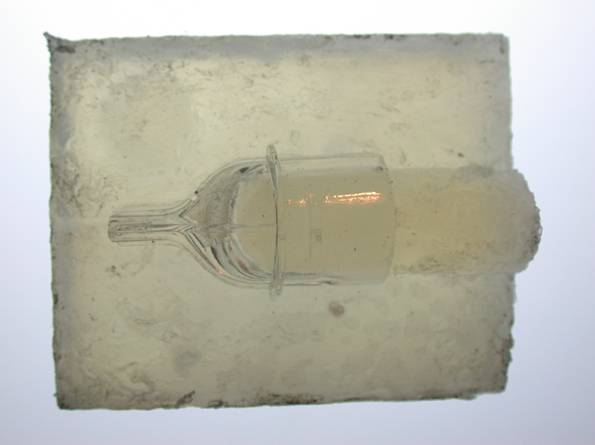


**b**

**a**

**d**

**c**

**Figure AF4**. RTV molds: a and b, mold for coupler (a, assembled mold; b, opened mold). c and d, mold for hub half (c,mold opened; d, portion of mold with cast of hub half in place).


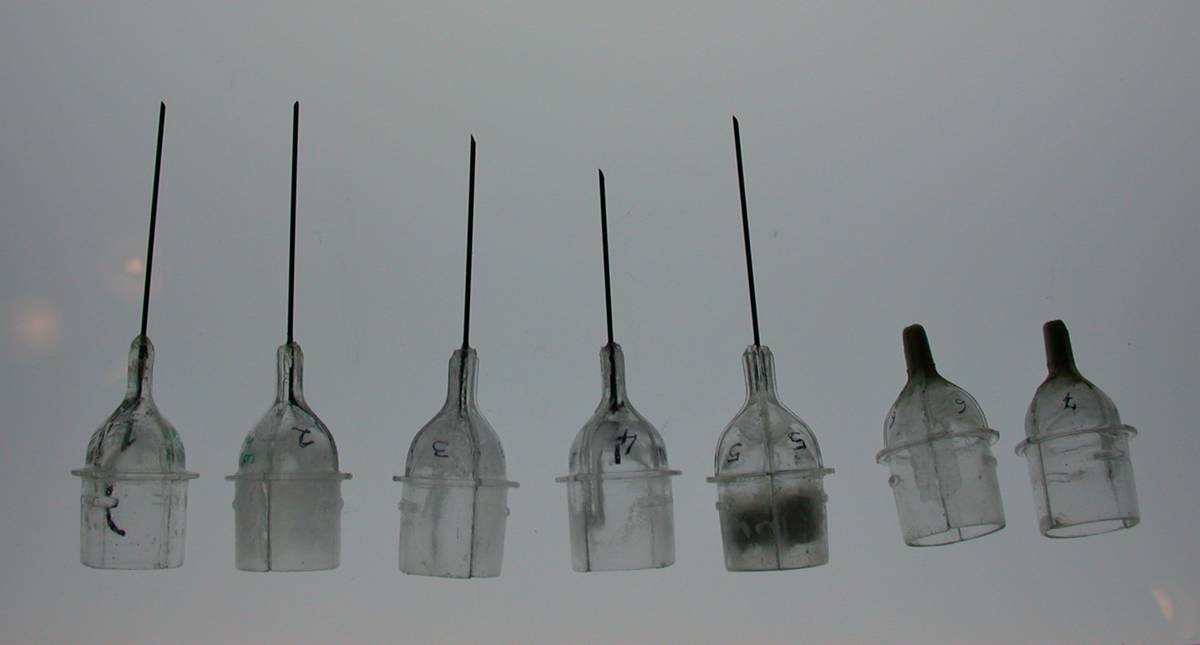


**1**

**2**

**3**

**4**

**5**

**6**

**7**

**Figure AF5**. Different types of needle modifications tested as pilot evaluation to select the final design(see Table AF1)
