# Supplementary material for: Tissue Harvester with Functional Valve (THFV): Shidham's device for reproducibly higher specimen yield by fine needle aspiration biopsy with easy to perform steps
Source: BMC Clin Pathol. 2007 Mar 7;7:2. doi: 10.1186/1472-6890-7-2 (PMC1829396; doi:10.1186/1472-6890-7-2)
Supplement: Additional File 2 — Schematic animation of sampling process with THFV. Animation demonstrating the sampling of tissue fragments with THFV. [file 1472-6890-7-2-S2.ppt]

## Slide 1
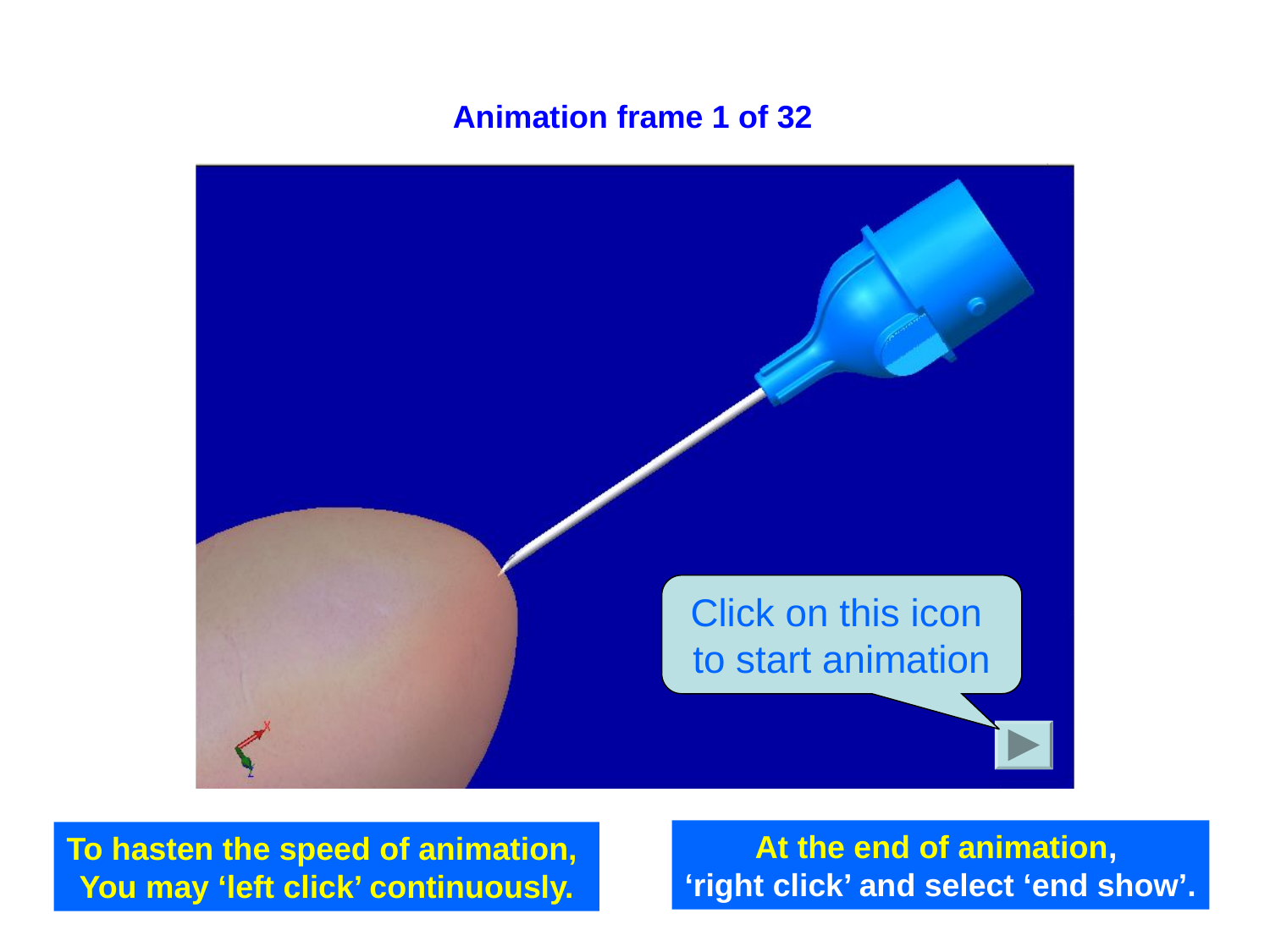

Animation frame 1 of 32
Click on this icon
to start animation
At the end of animation,
‘right click’ and select ‘end show’.
To hasten the speed of animation,
You may ‘left click’ continuously.

## Slide 2
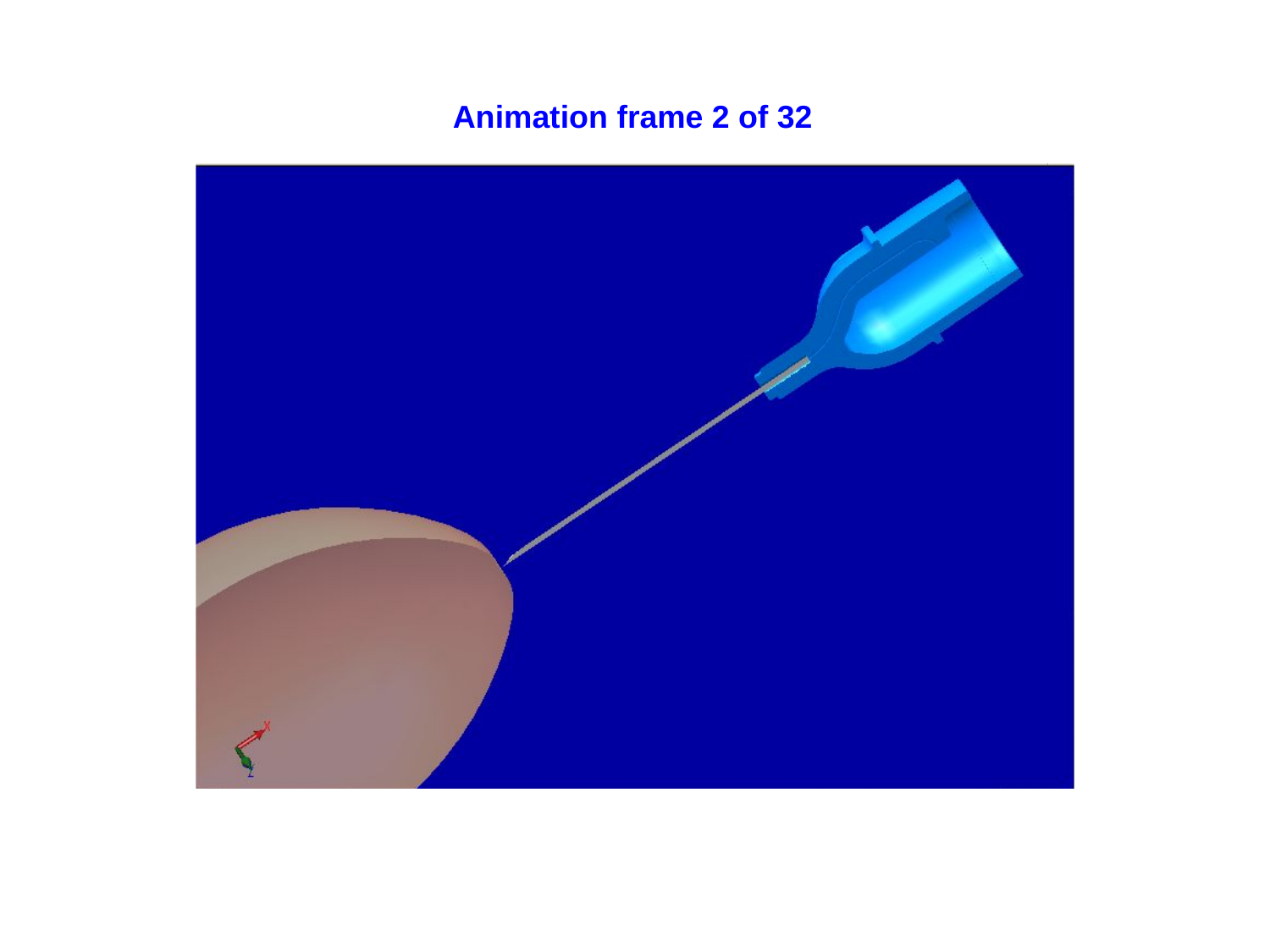

Animation frame 2 of 32

## Slide 3
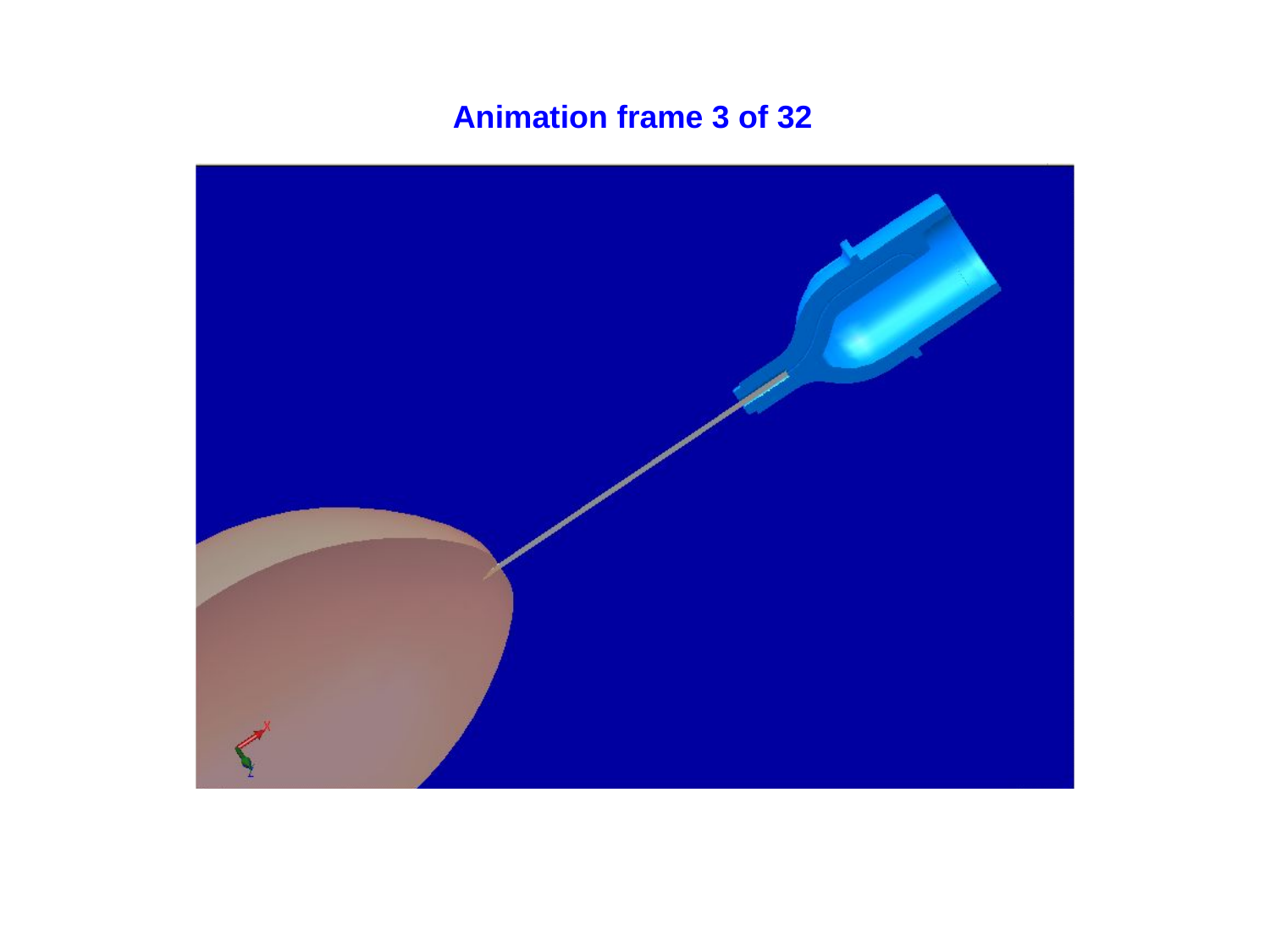

Animation frame 3 of 32

## Slide 4
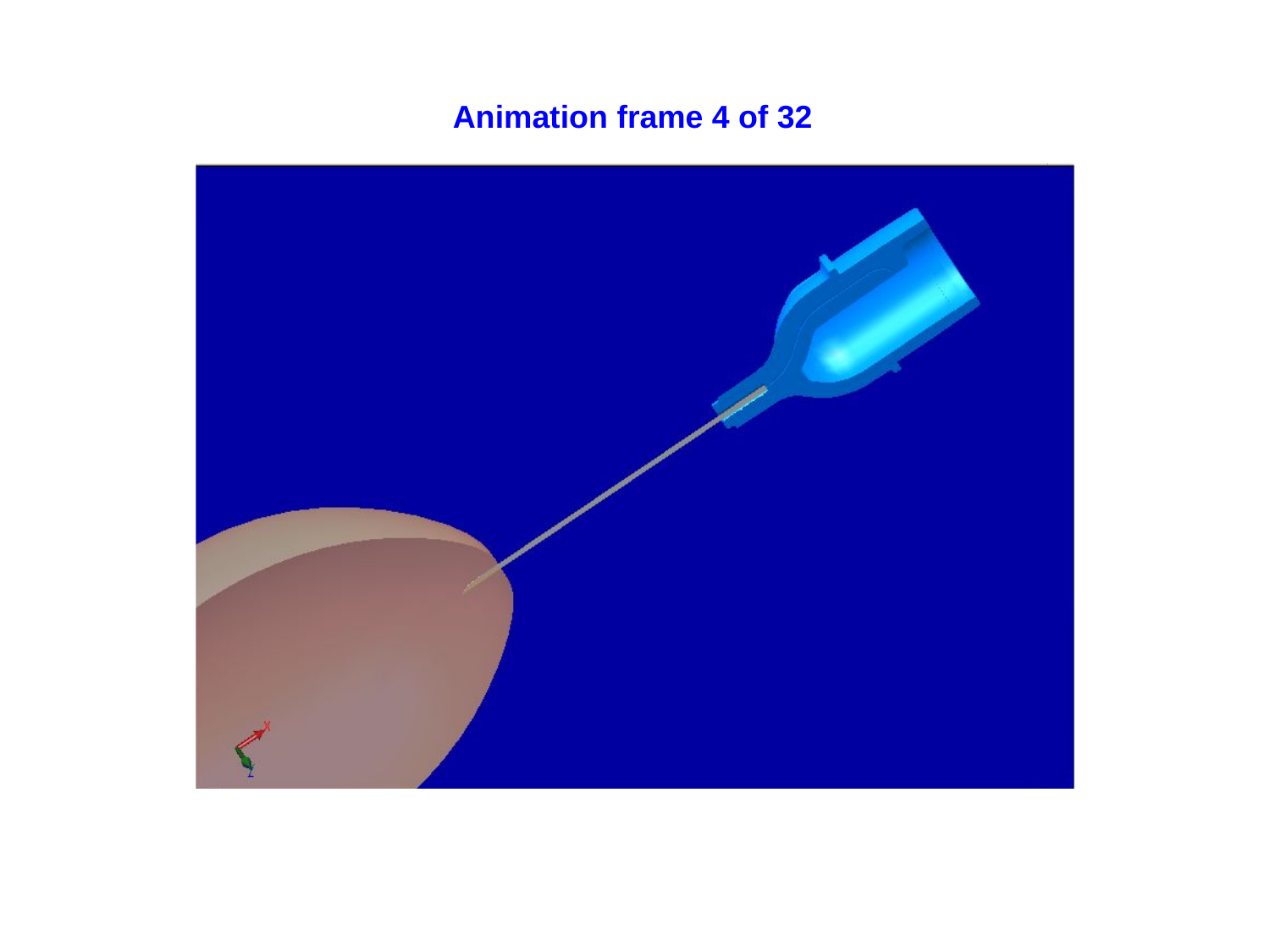

Animation frame 4 of 32

## Slide 5
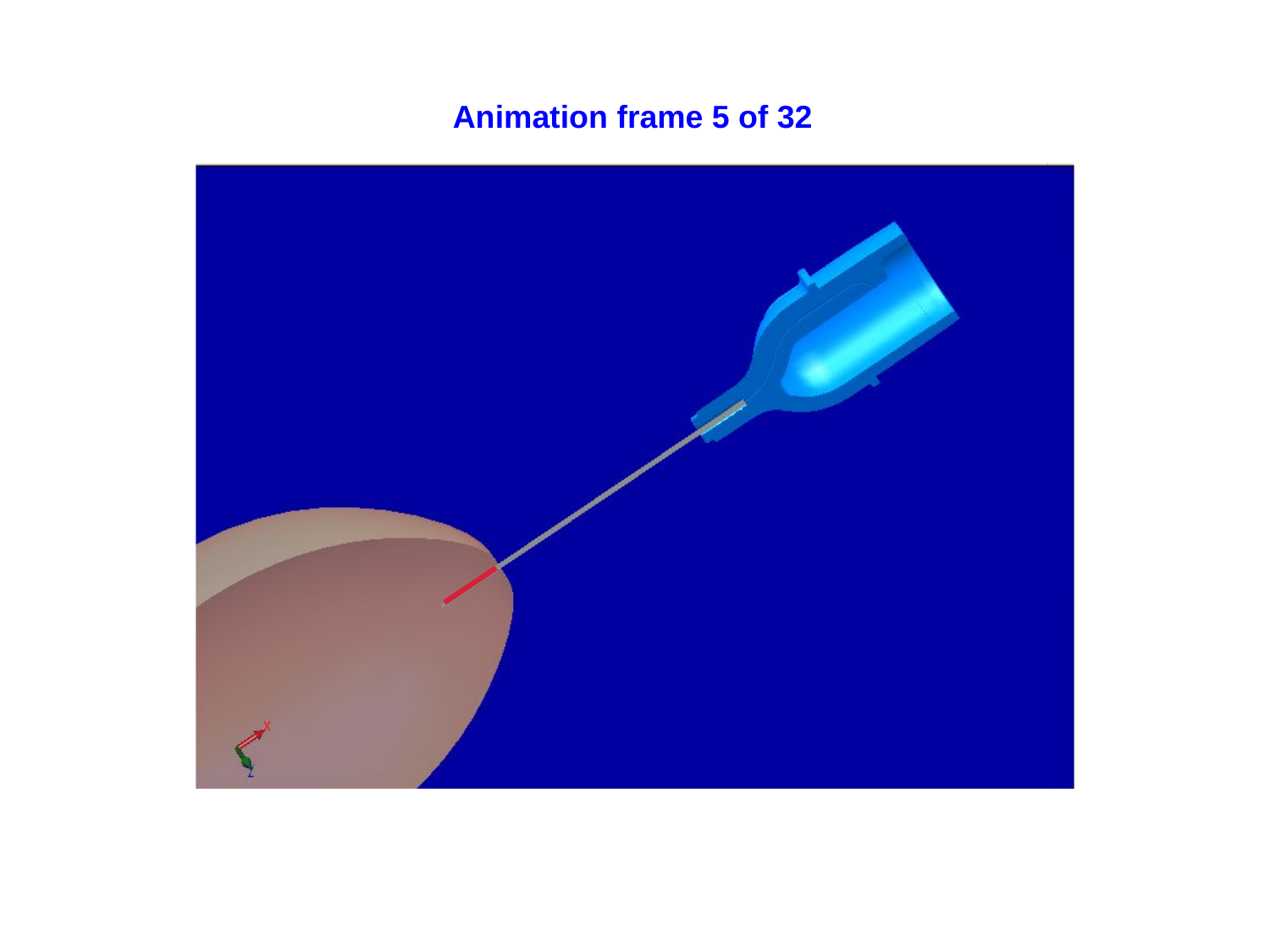

Animation frame 5 of 32

## Slide 6
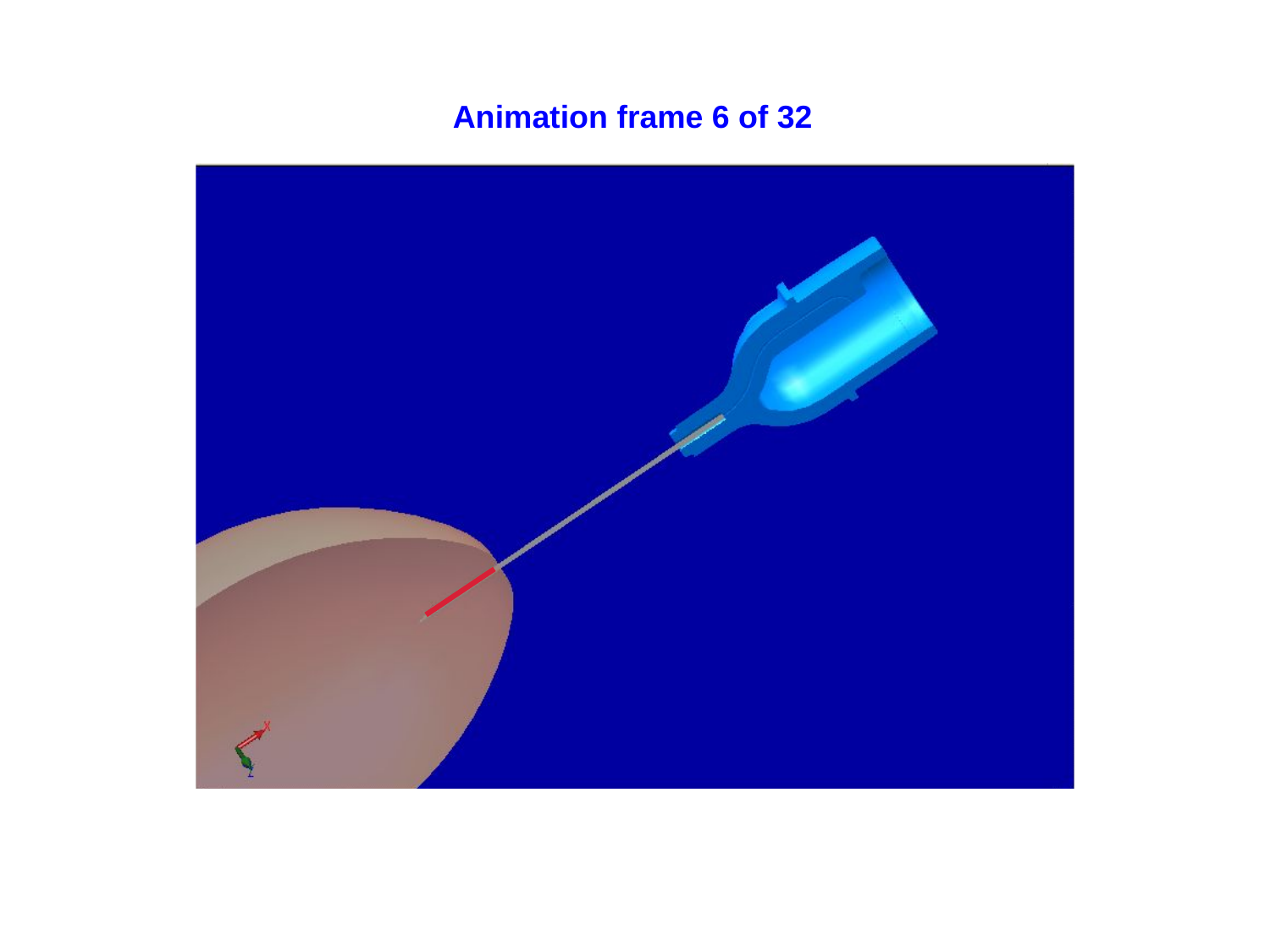

Animation frame 6 of 32

## Slide 7
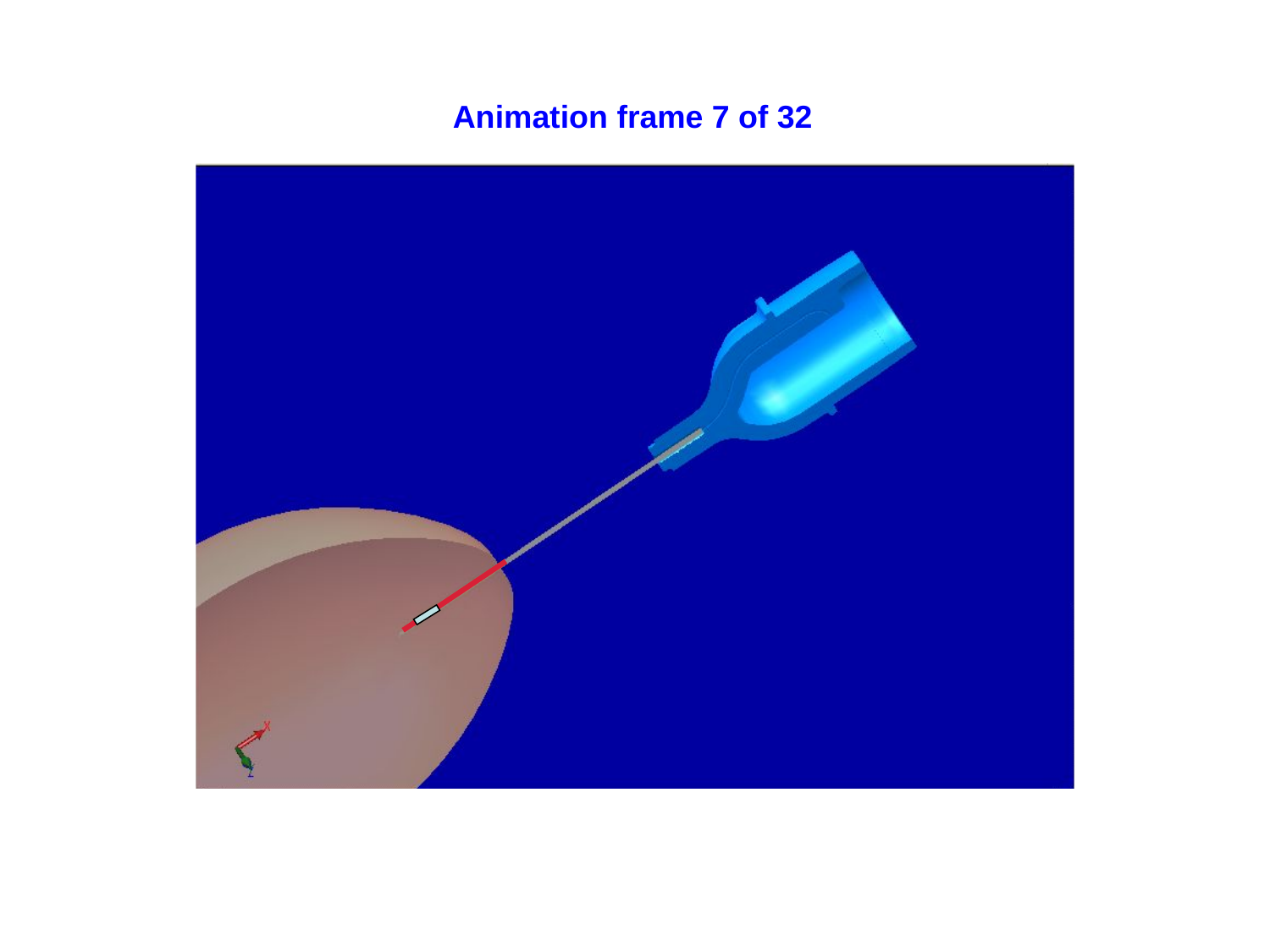

Animation frame 7 of 32

## Slide 8
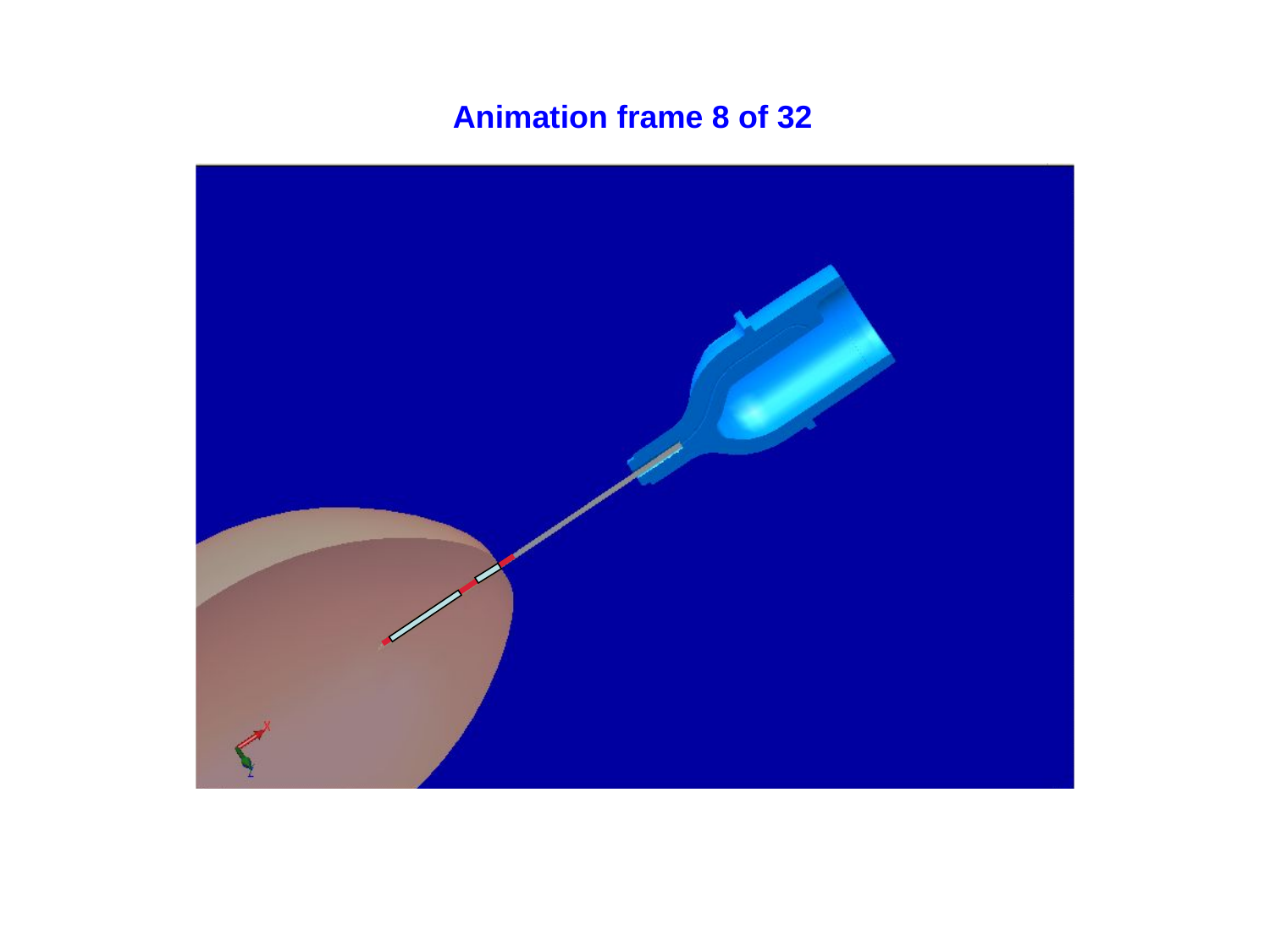

Animation frame 8 of 32

## Slide 9
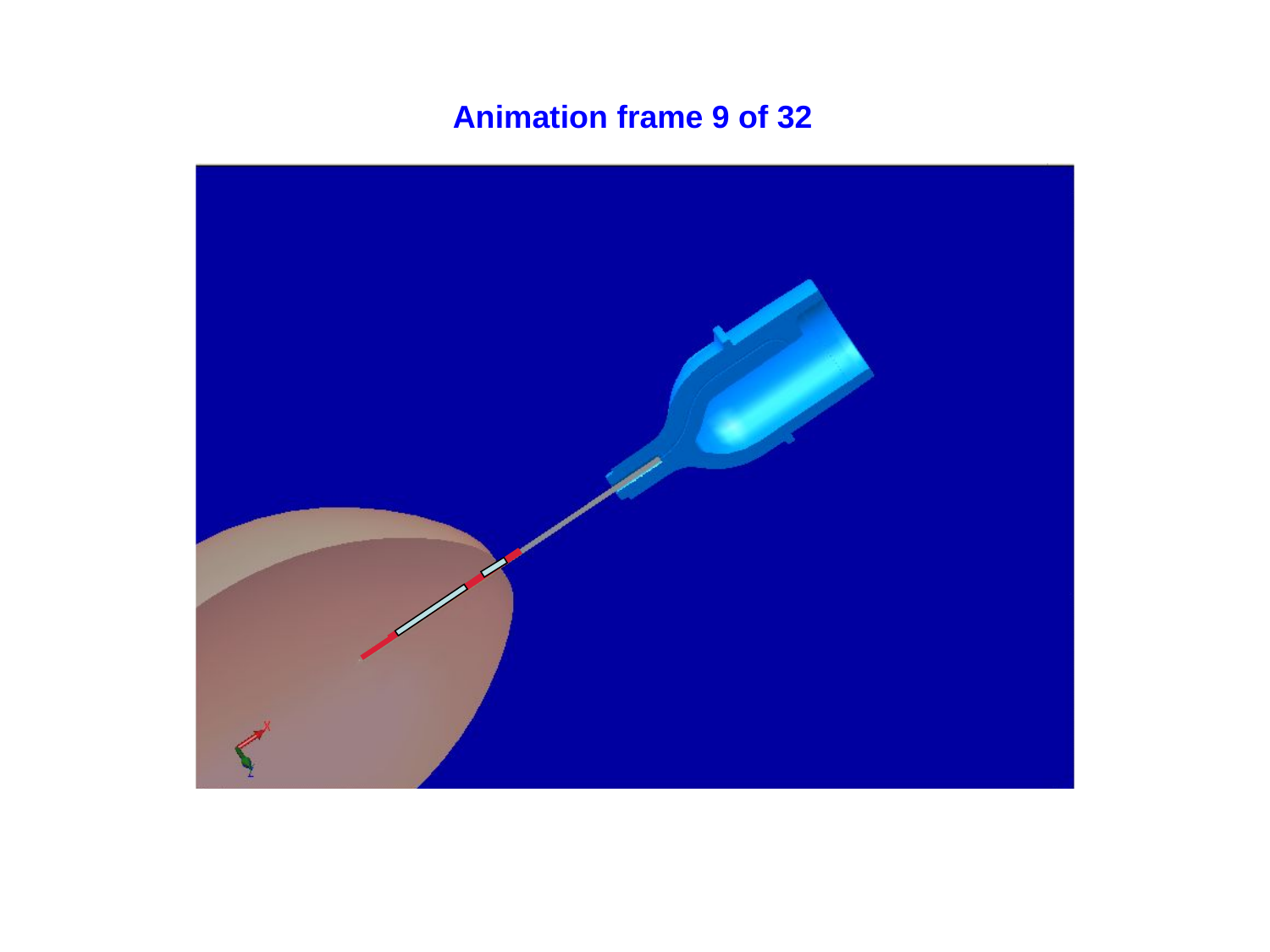

Animation frame 9 of 32

## Slide 10
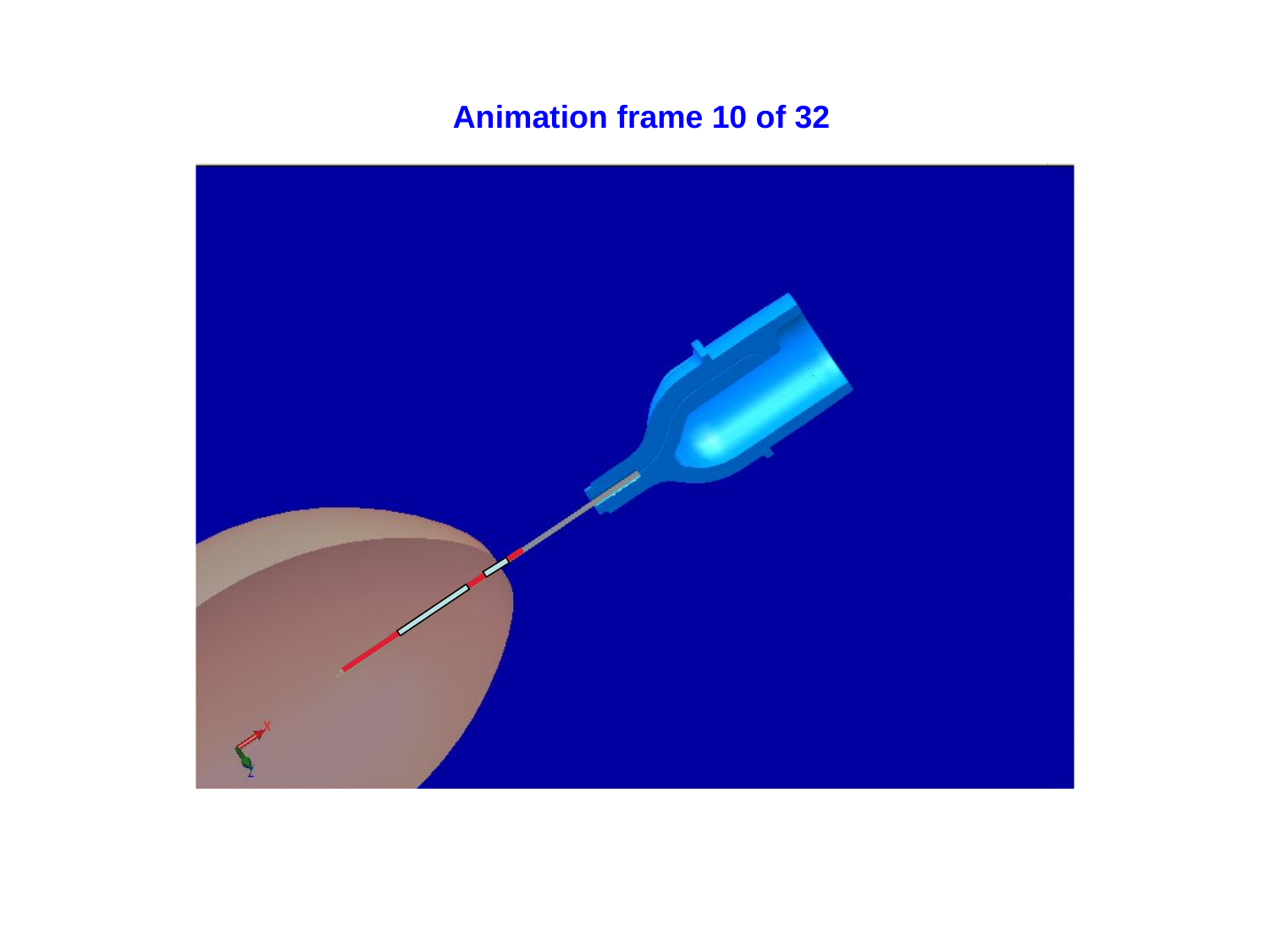

Animation frame 10 of 32

## Slide 11
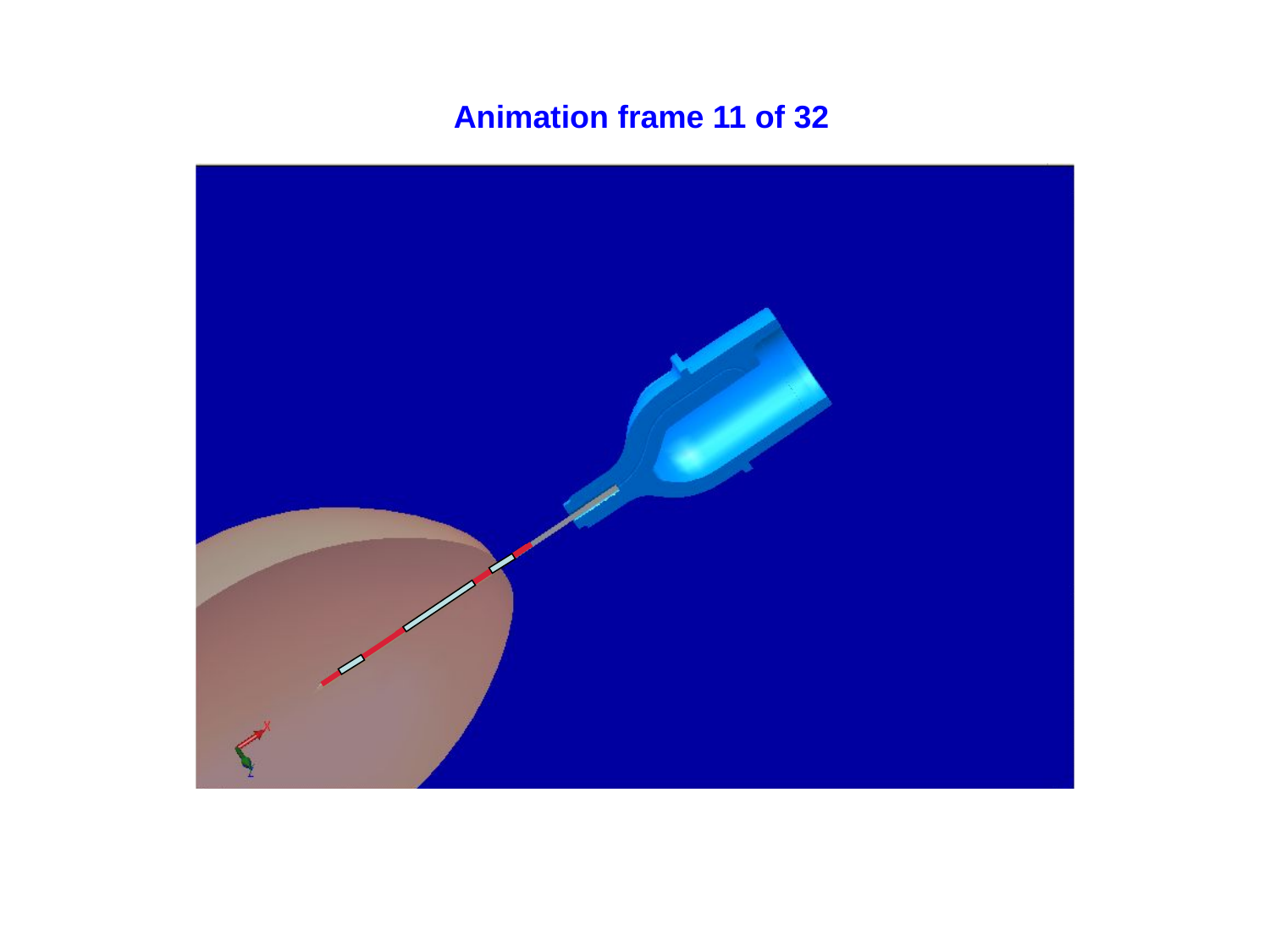

Animation frame 11 of 32

## Slide 12
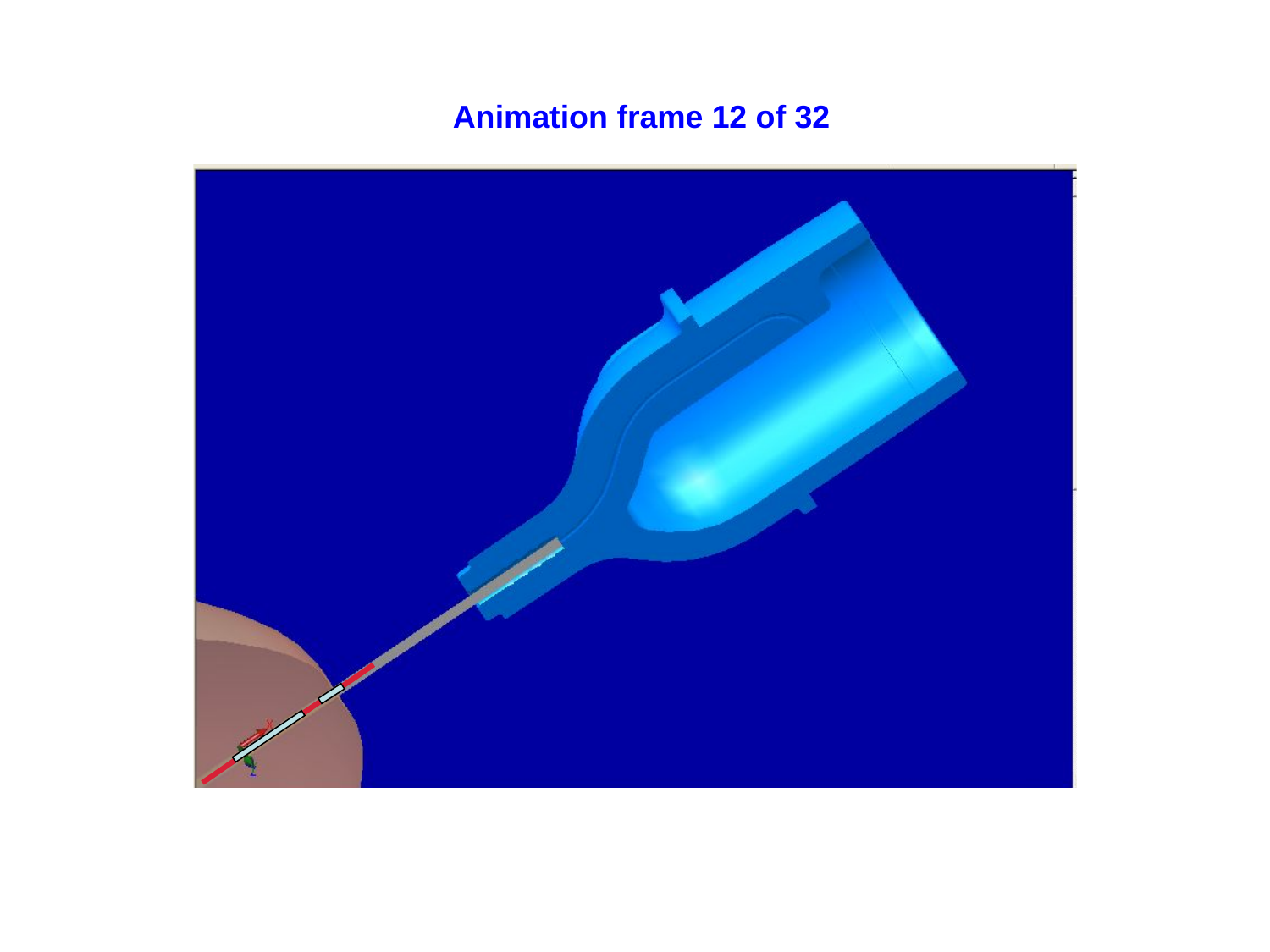

Animation frame 12 of 32

## Slide 13
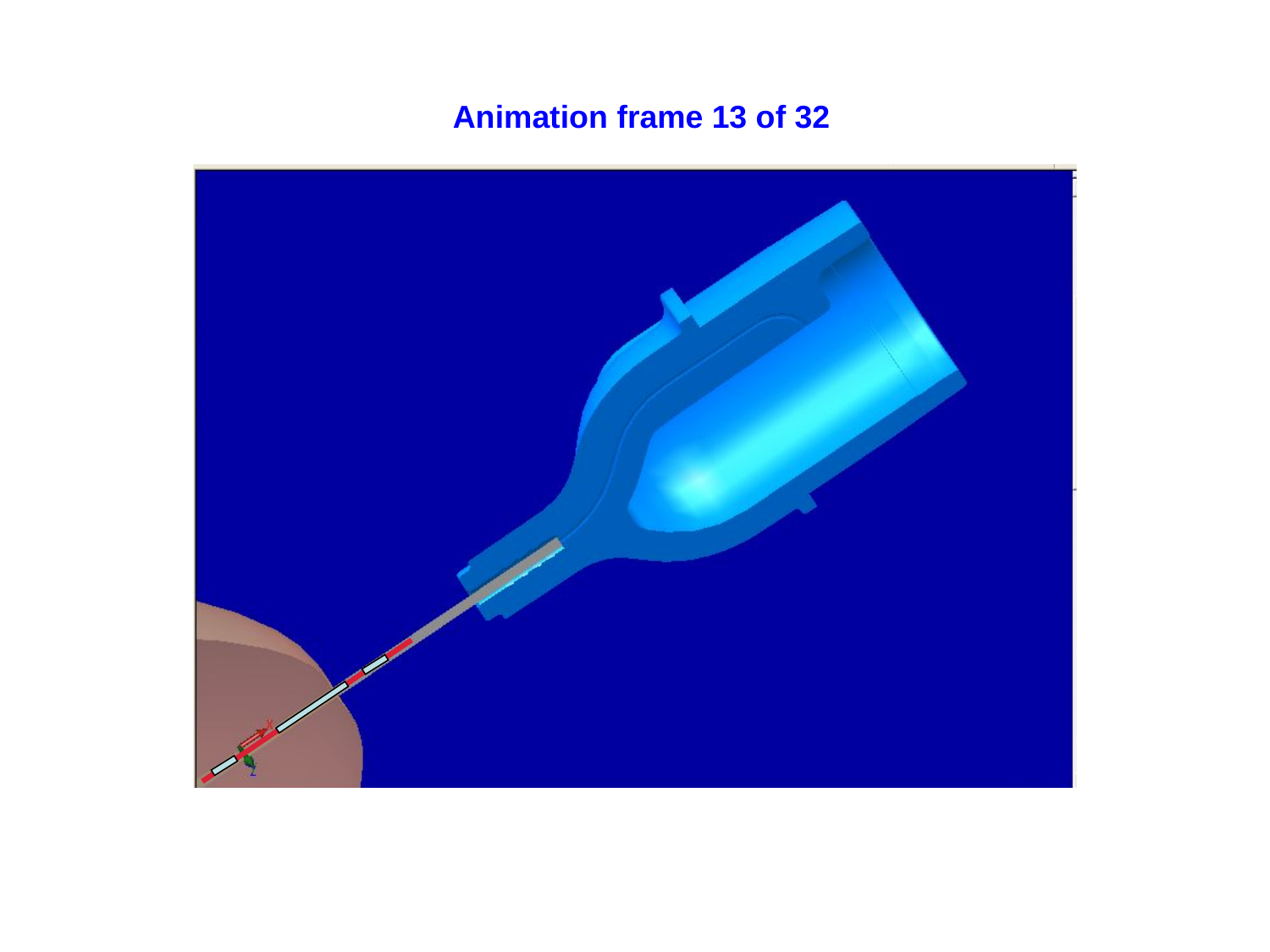

Animation frame 13 of 32

## Slide 14
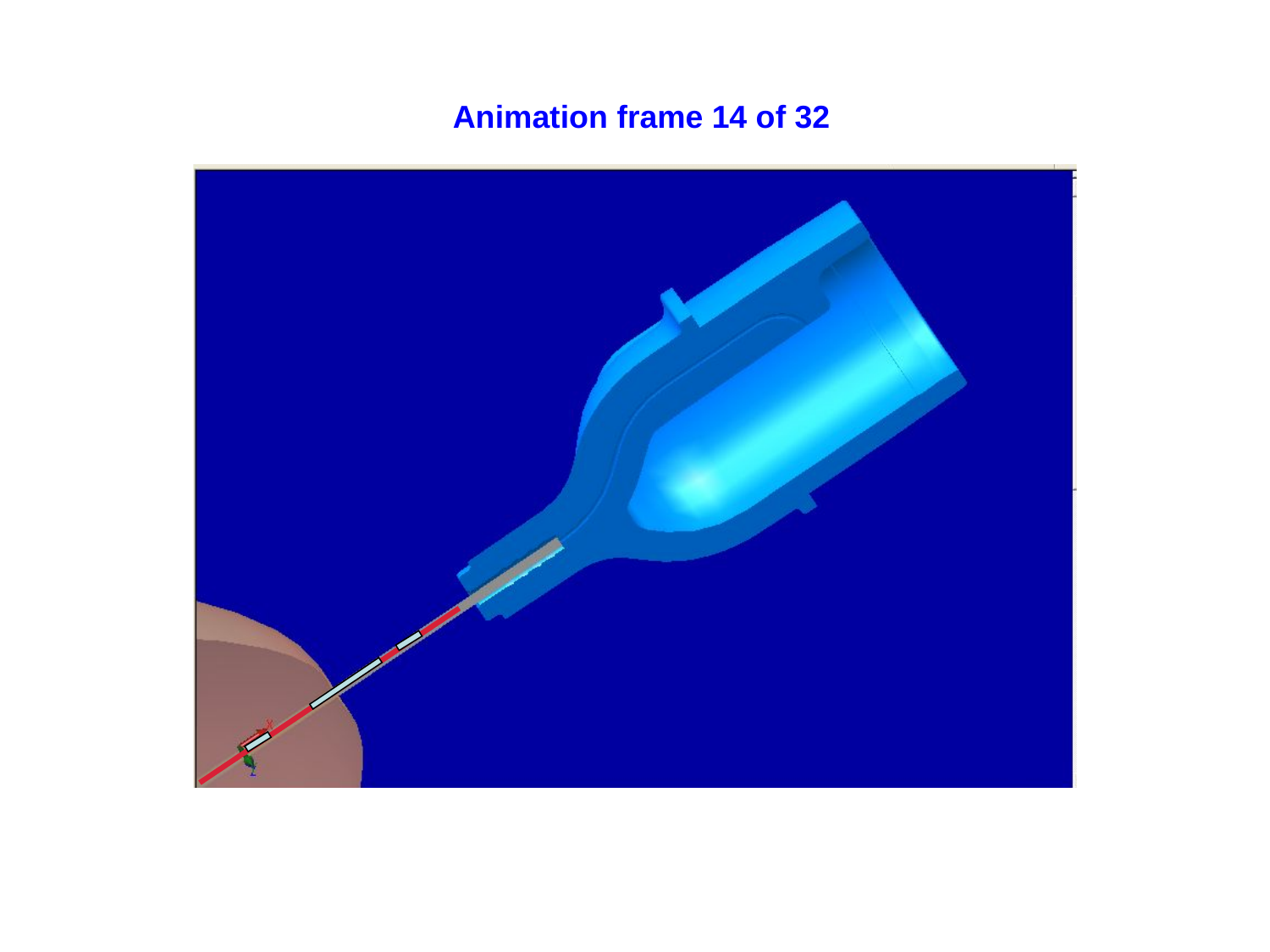

Animation frame 14 of 32

## Slide 15
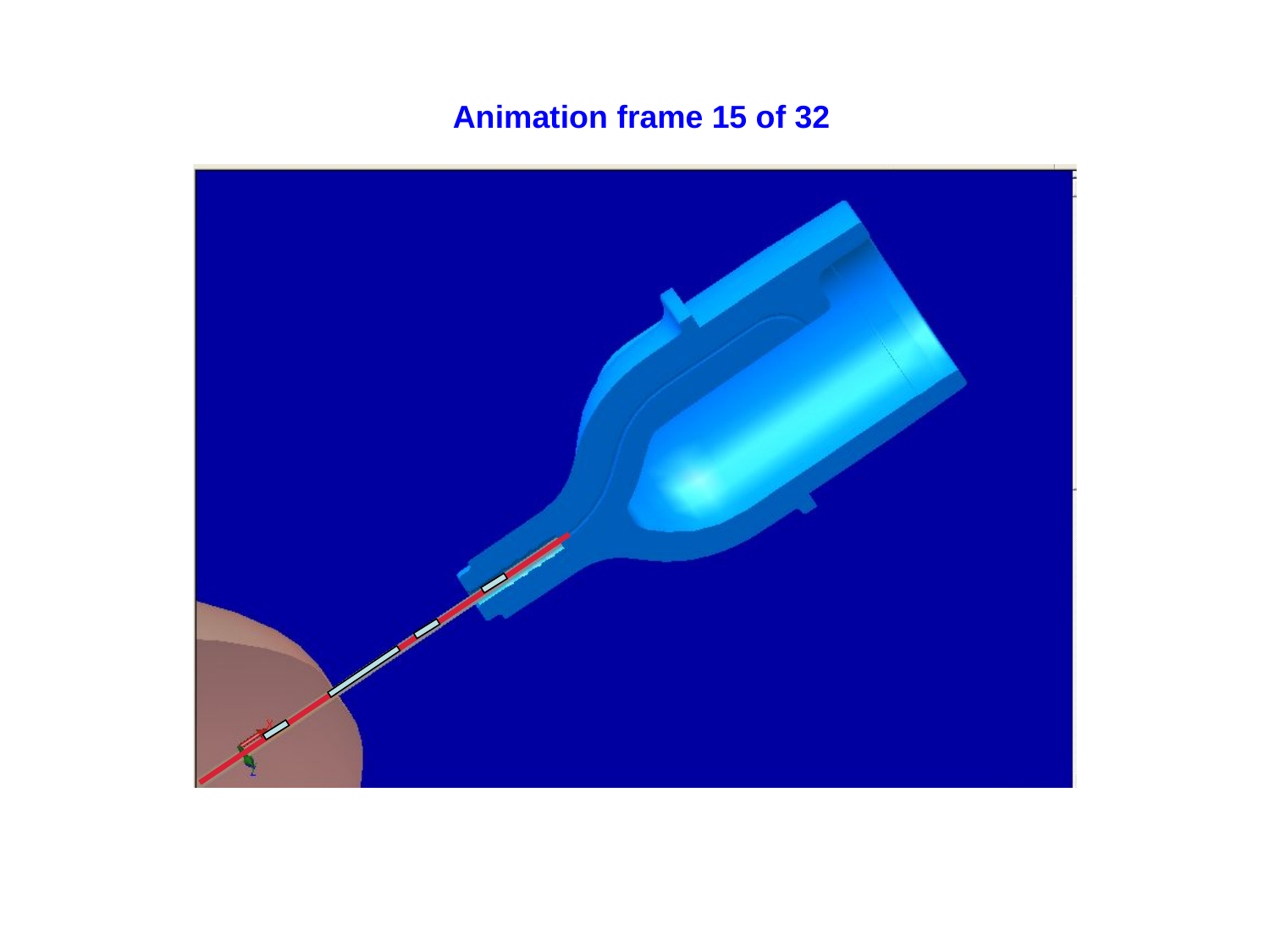

Animation frame 15 of 32

## Slide 16
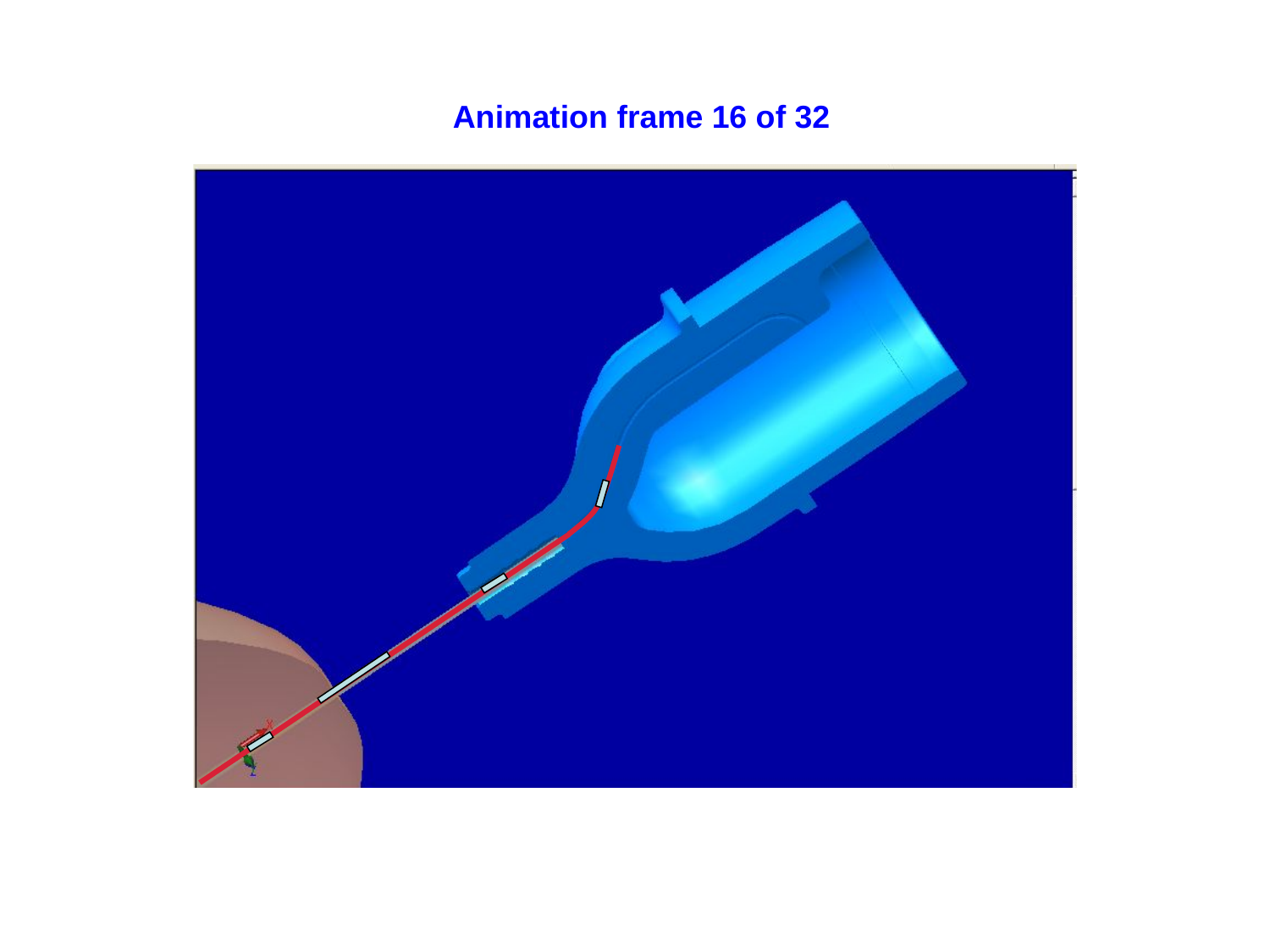

Animation frame 16 of 32

## Slide 17
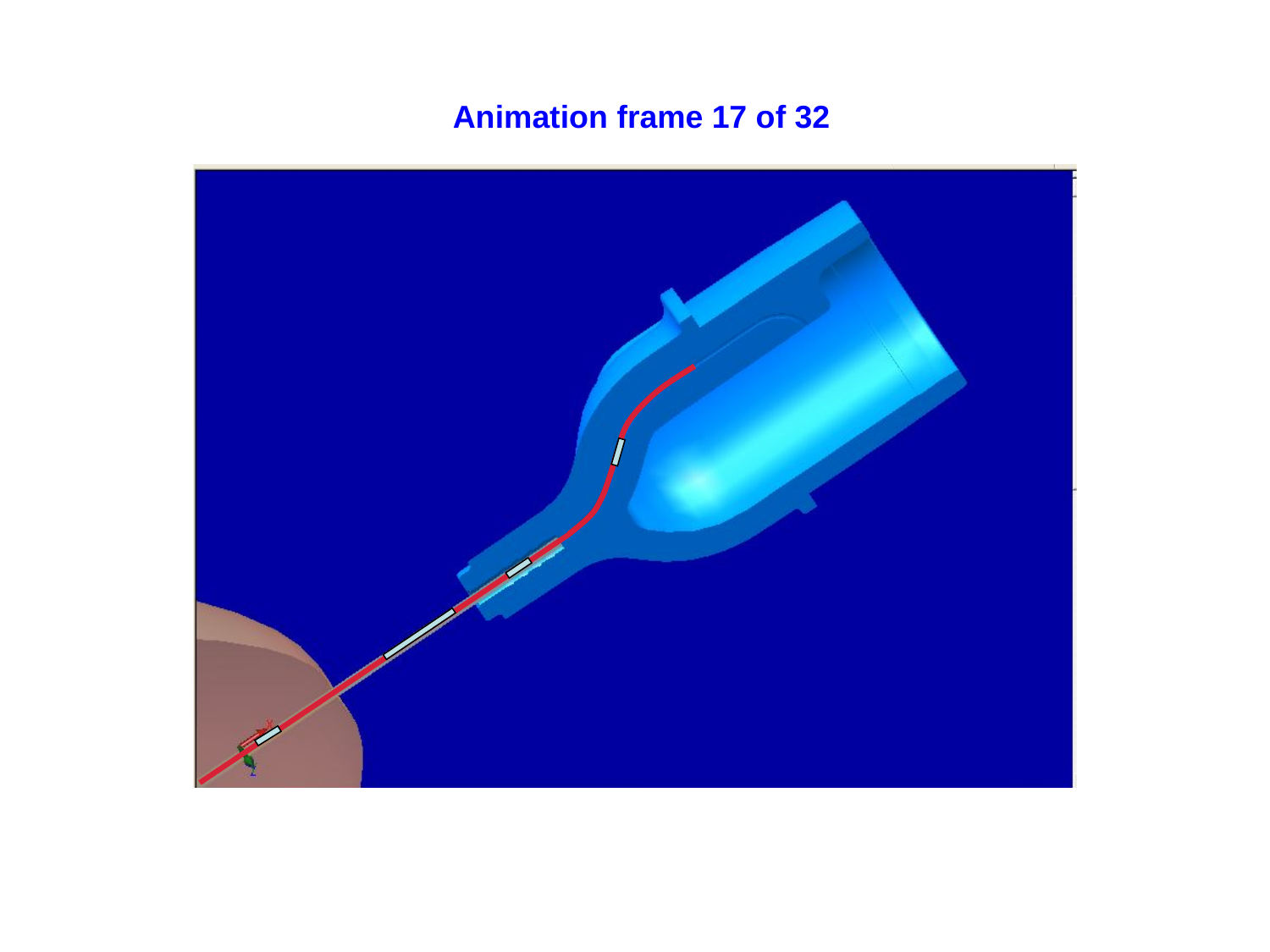

Animation frame 17 of 32

## Slide 18
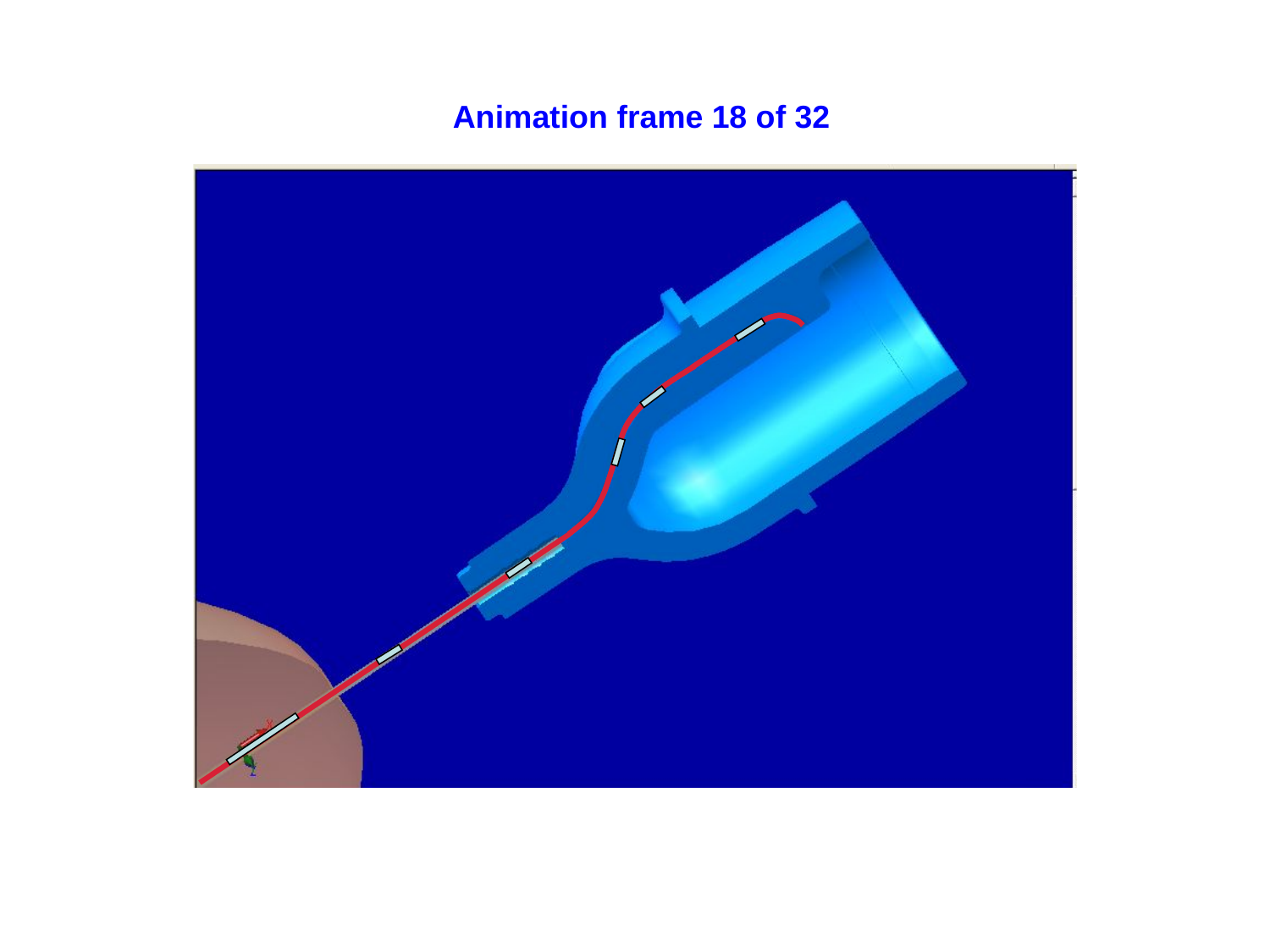

Animation frame 18 of 32

## Slide 19
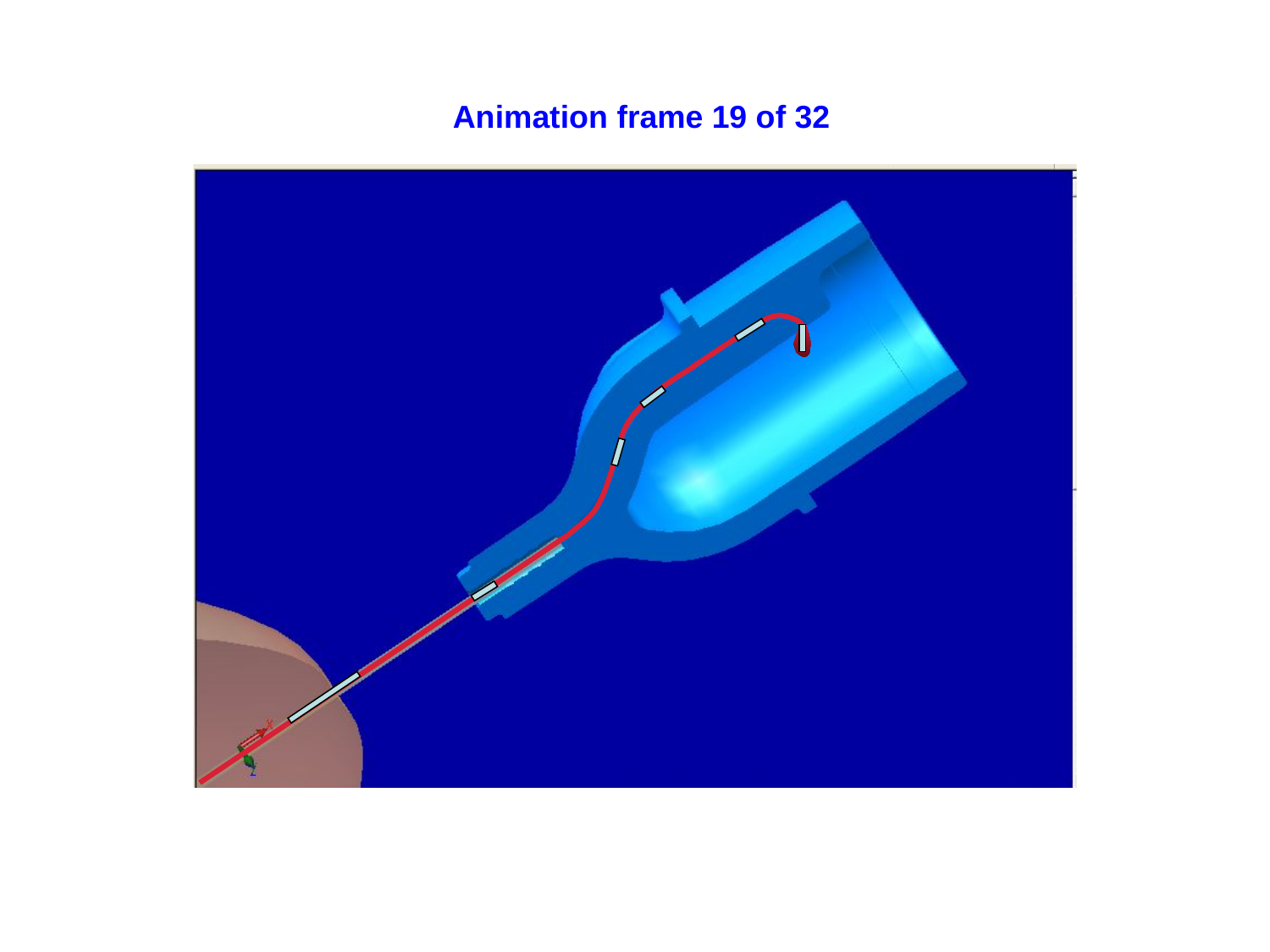

Animation frame 19 of 32

## Slide 20
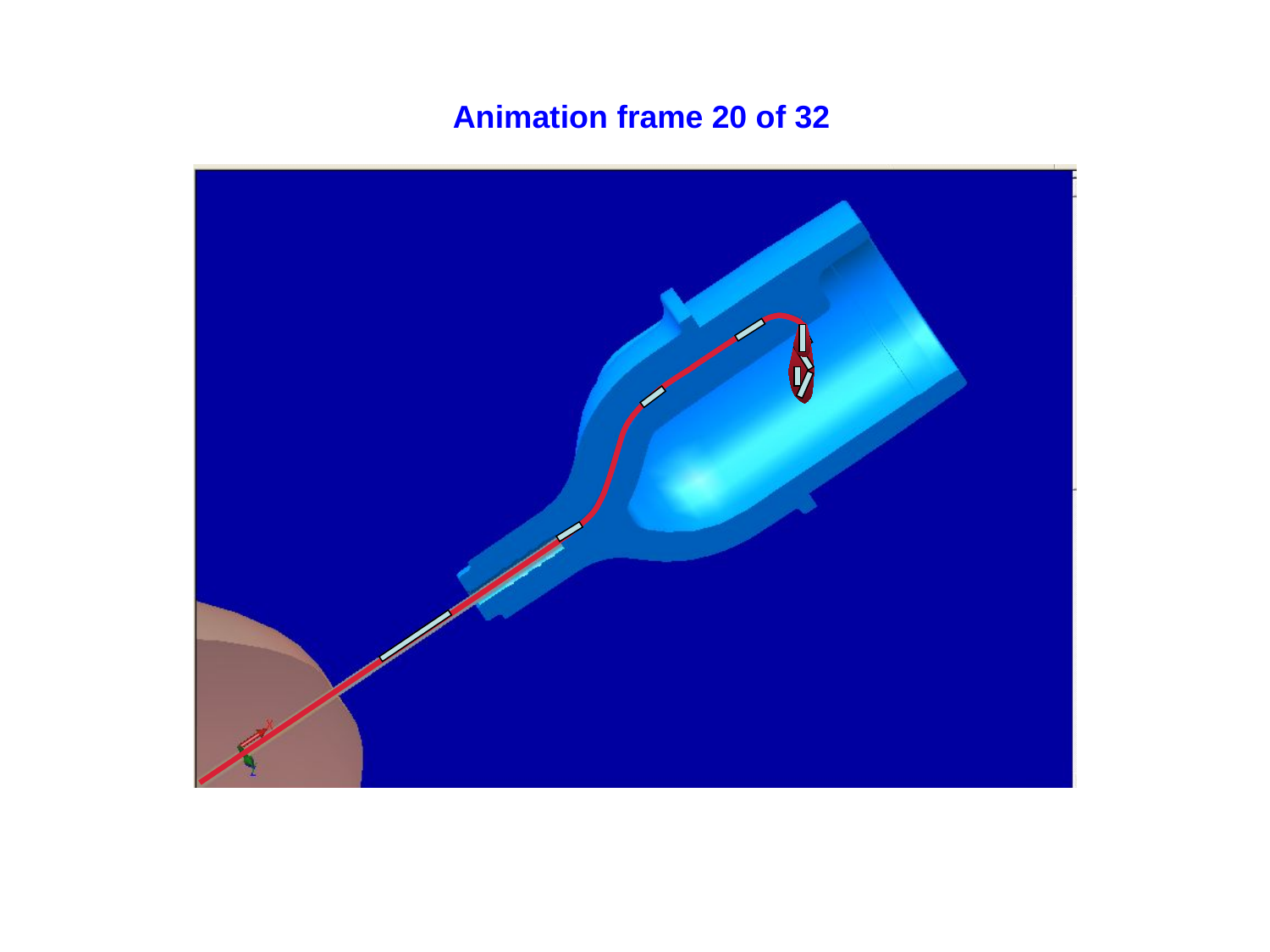

Animation frame 20 of 32

## Slide 21
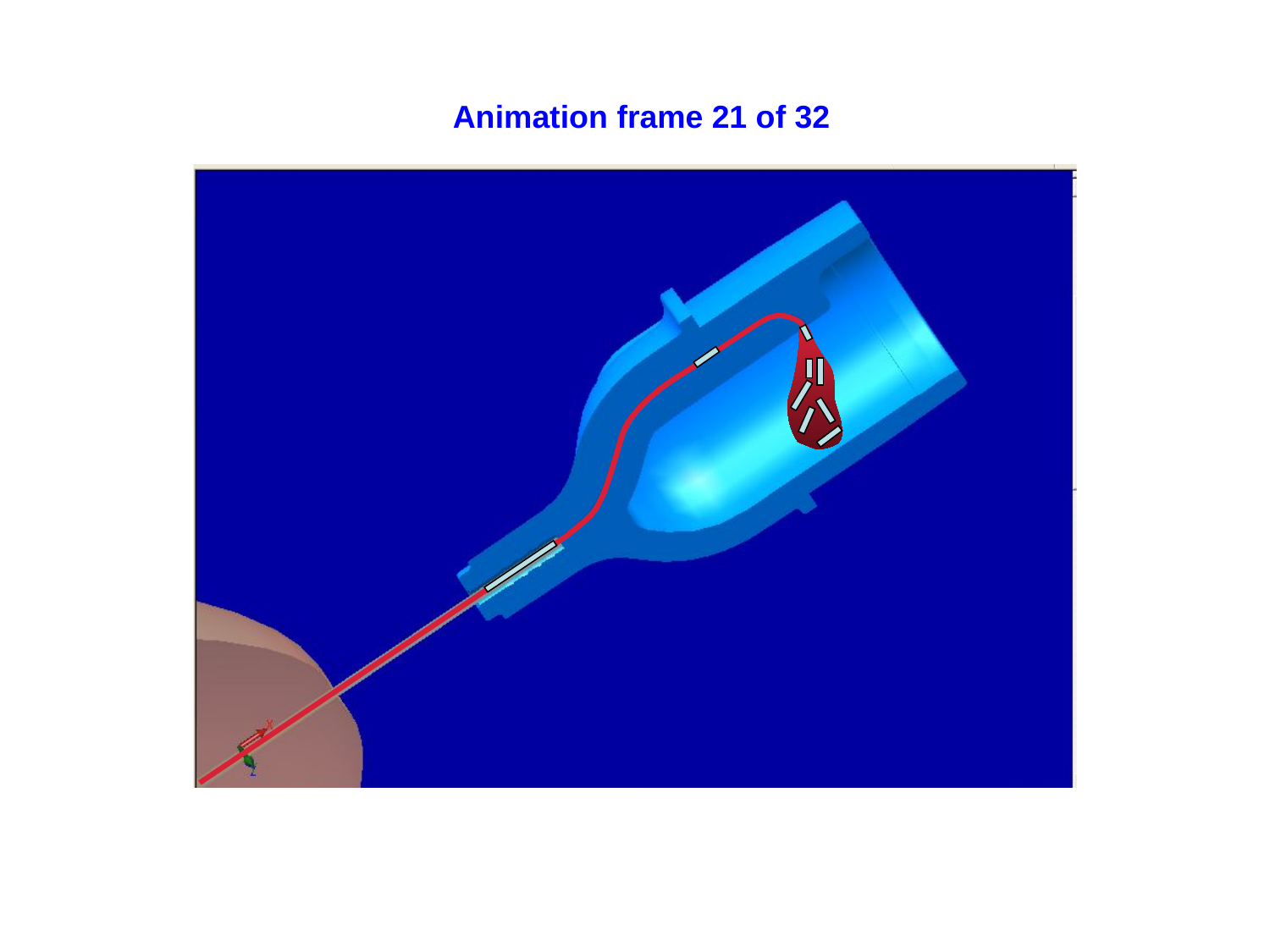

Animation frame 21 of 32

## Slide 22
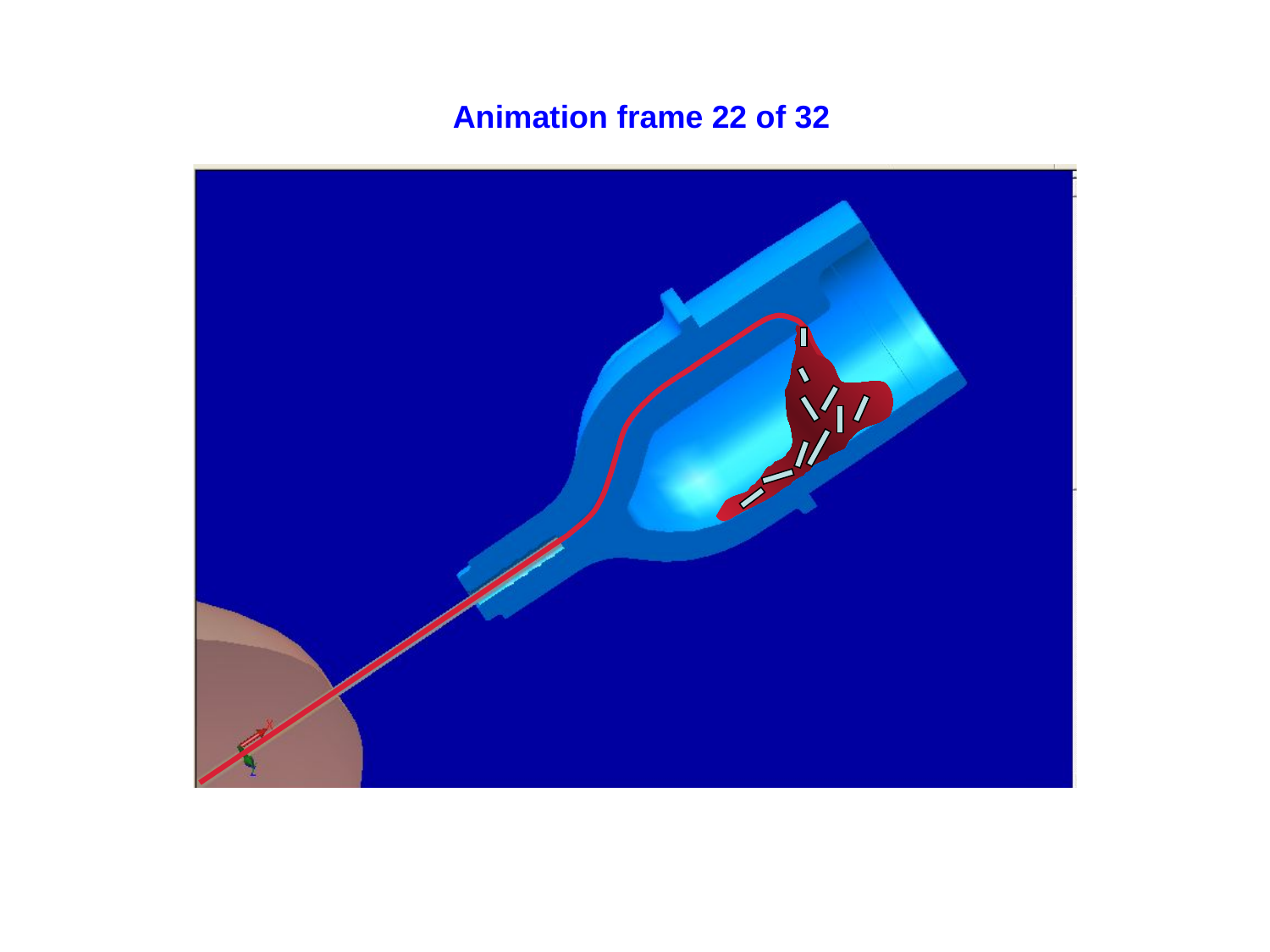

Animation frame 22 of 32

## Slide 23
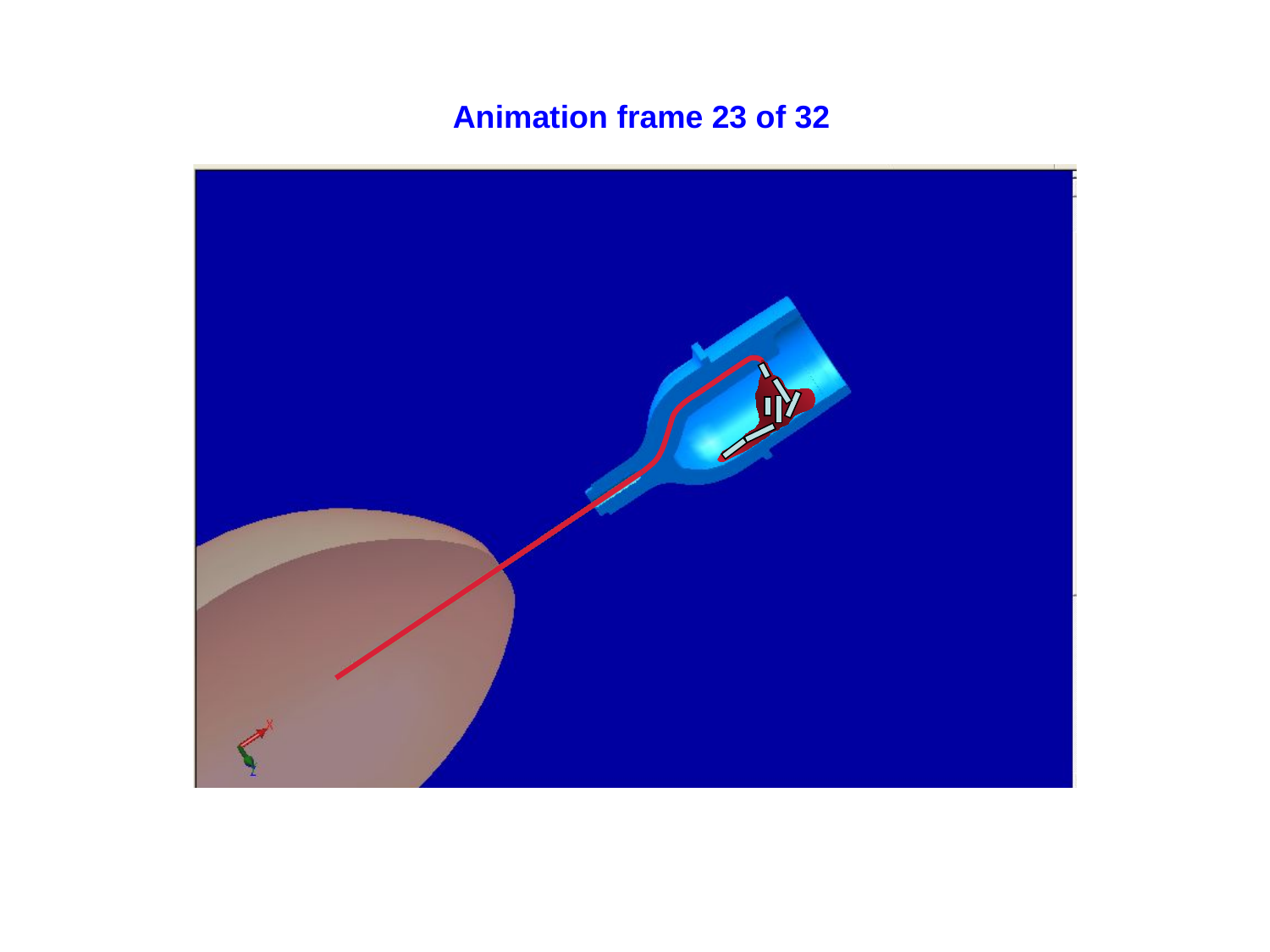

Animation frame 23 of 32

## Slide 24
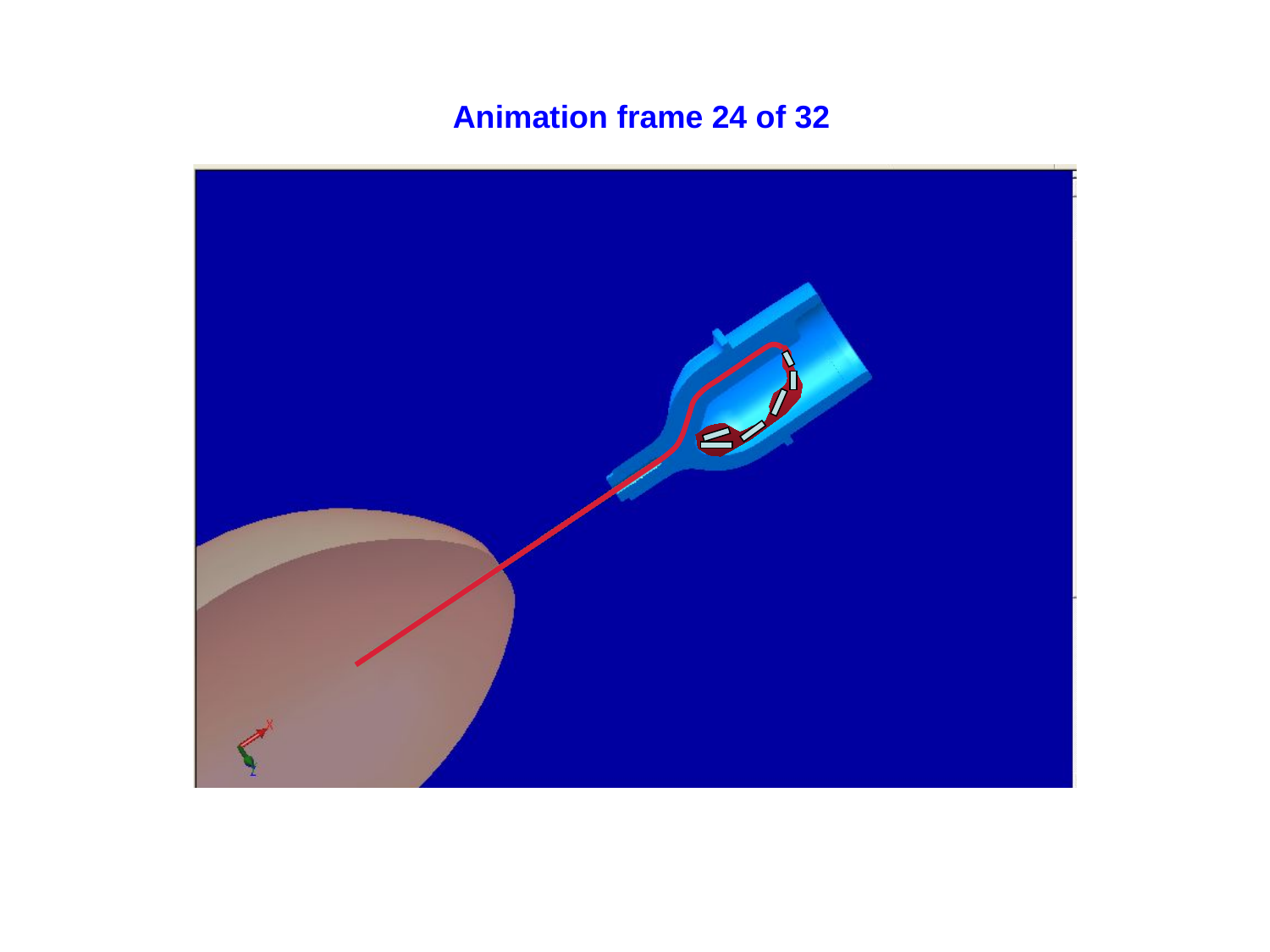

Animation frame 24 of 32

## Slide 25
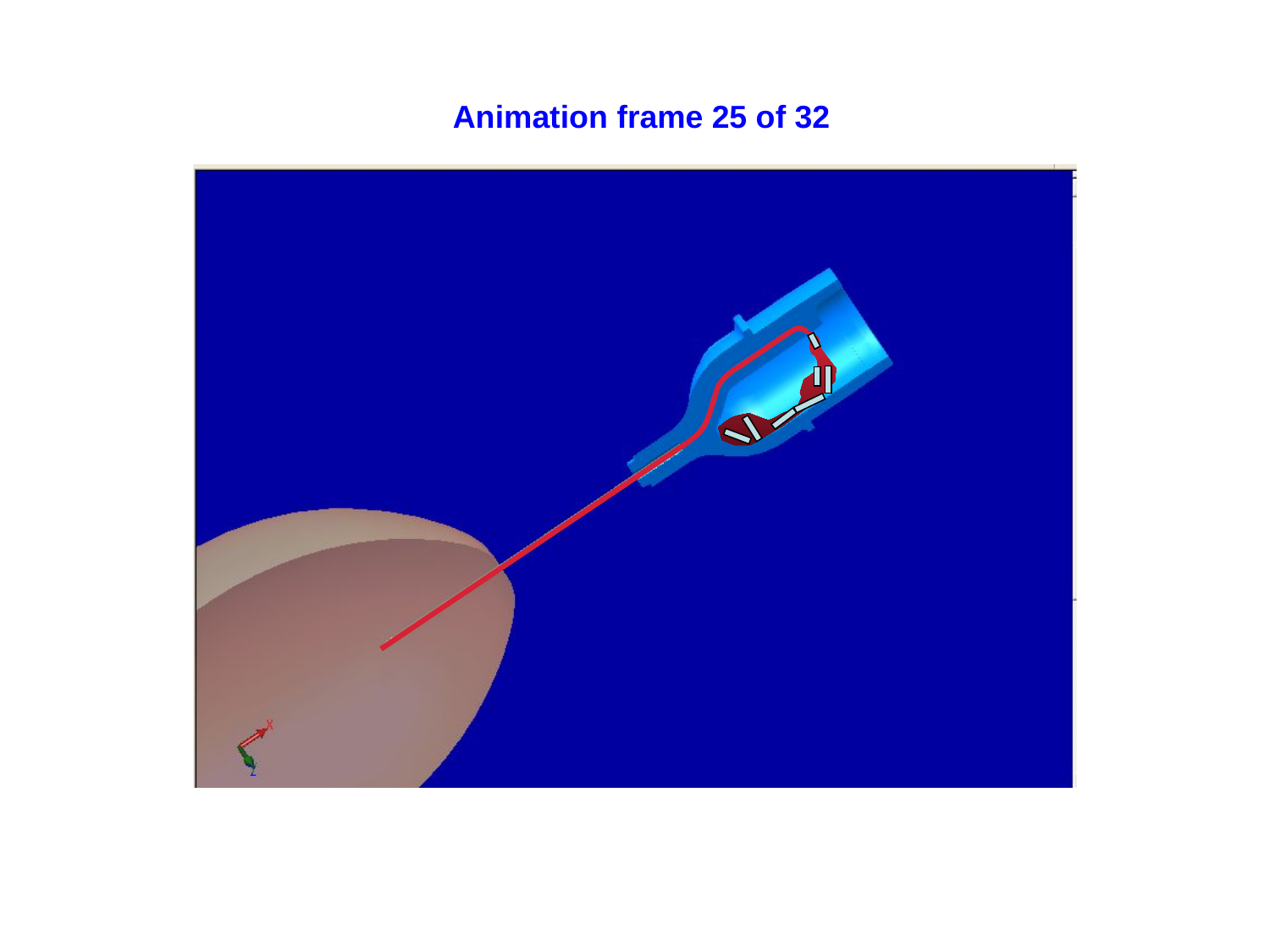

Animation frame 25 of 32

## Slide 26
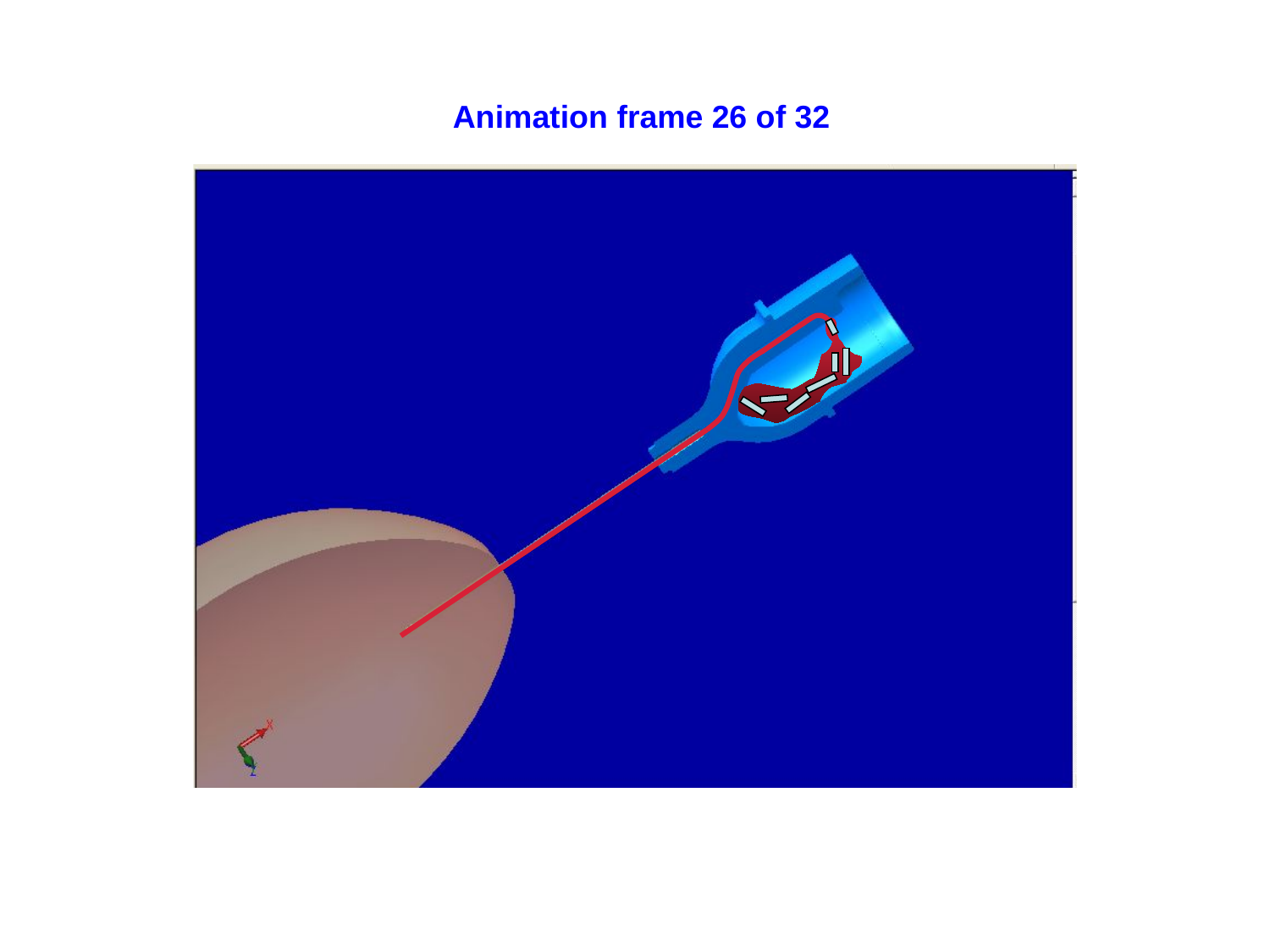

Animation frame 26 of 32

## Slide 27
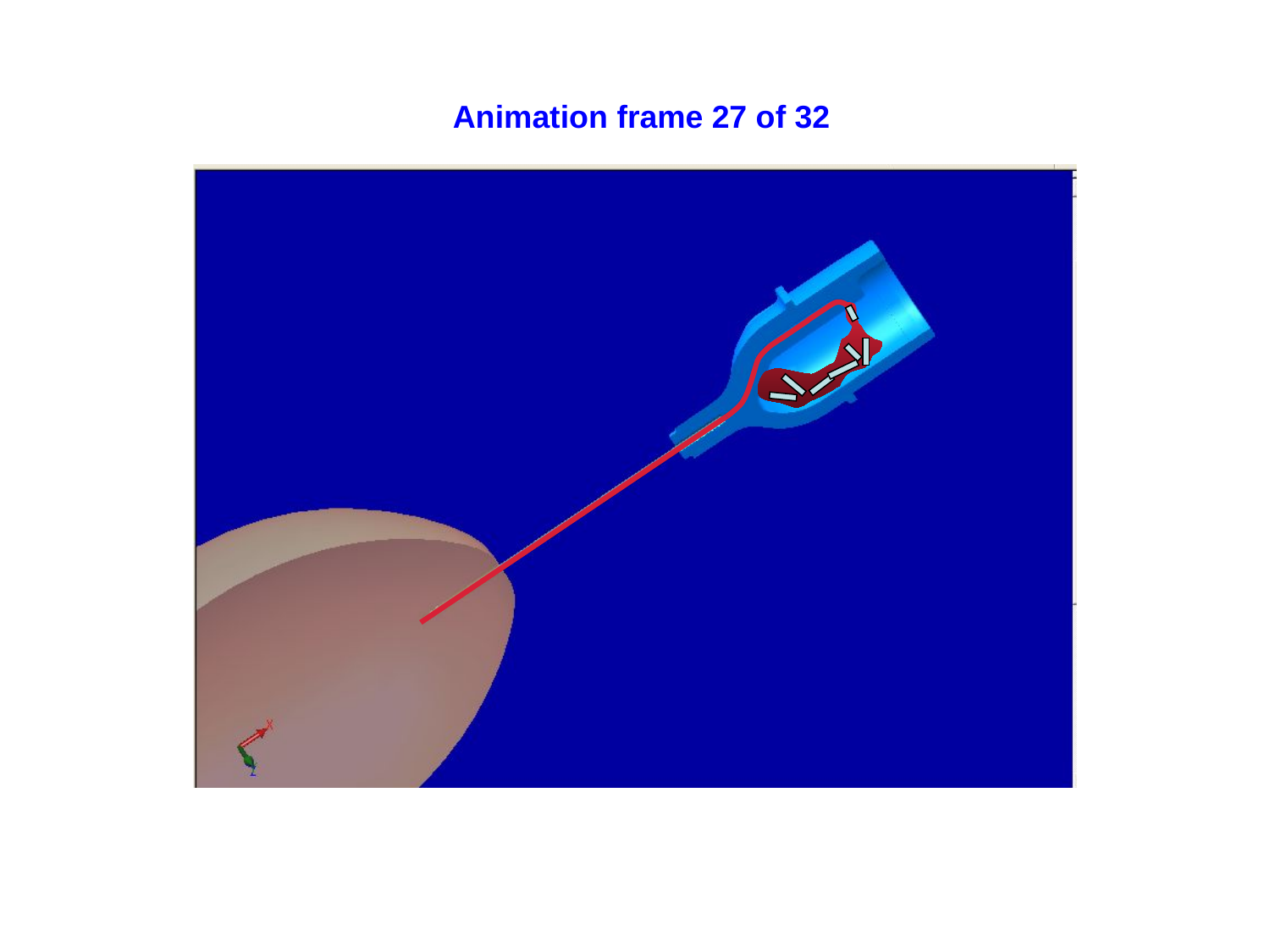

Animation frame 27 of 32

## Slide 28
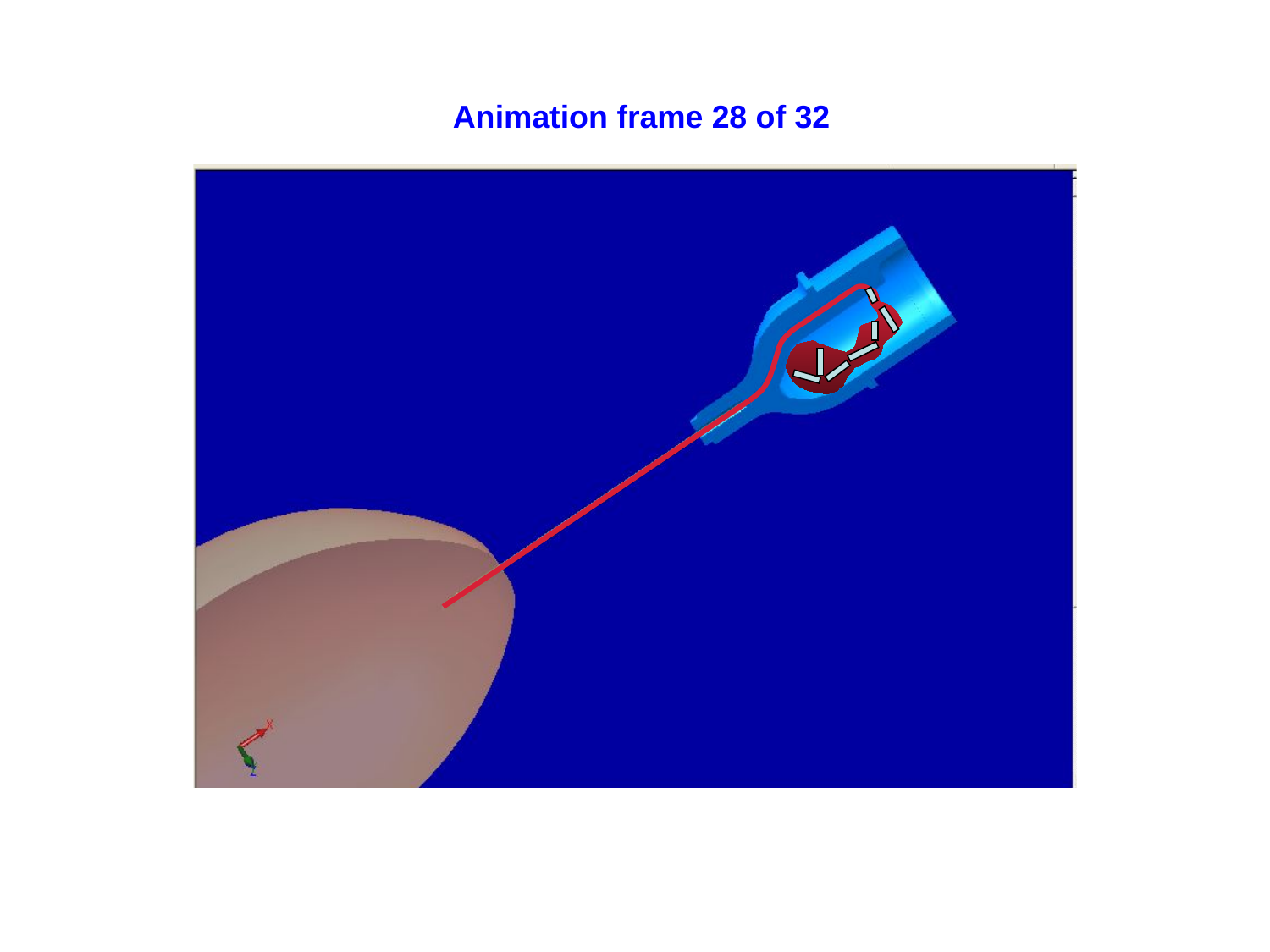

Animation frame 28 of 32

## Slide 29
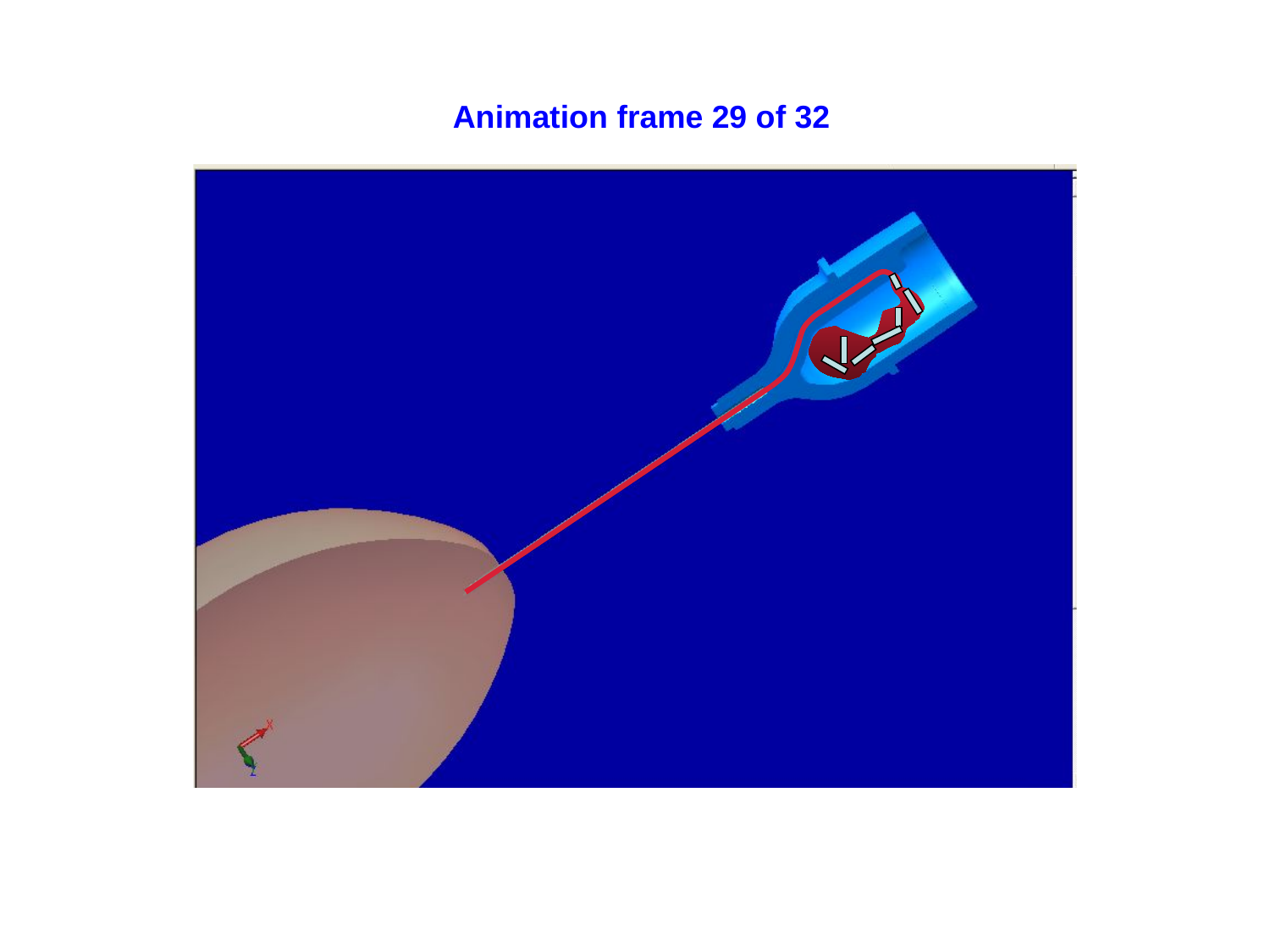

Animation frame 29 of 32

## Slide 30
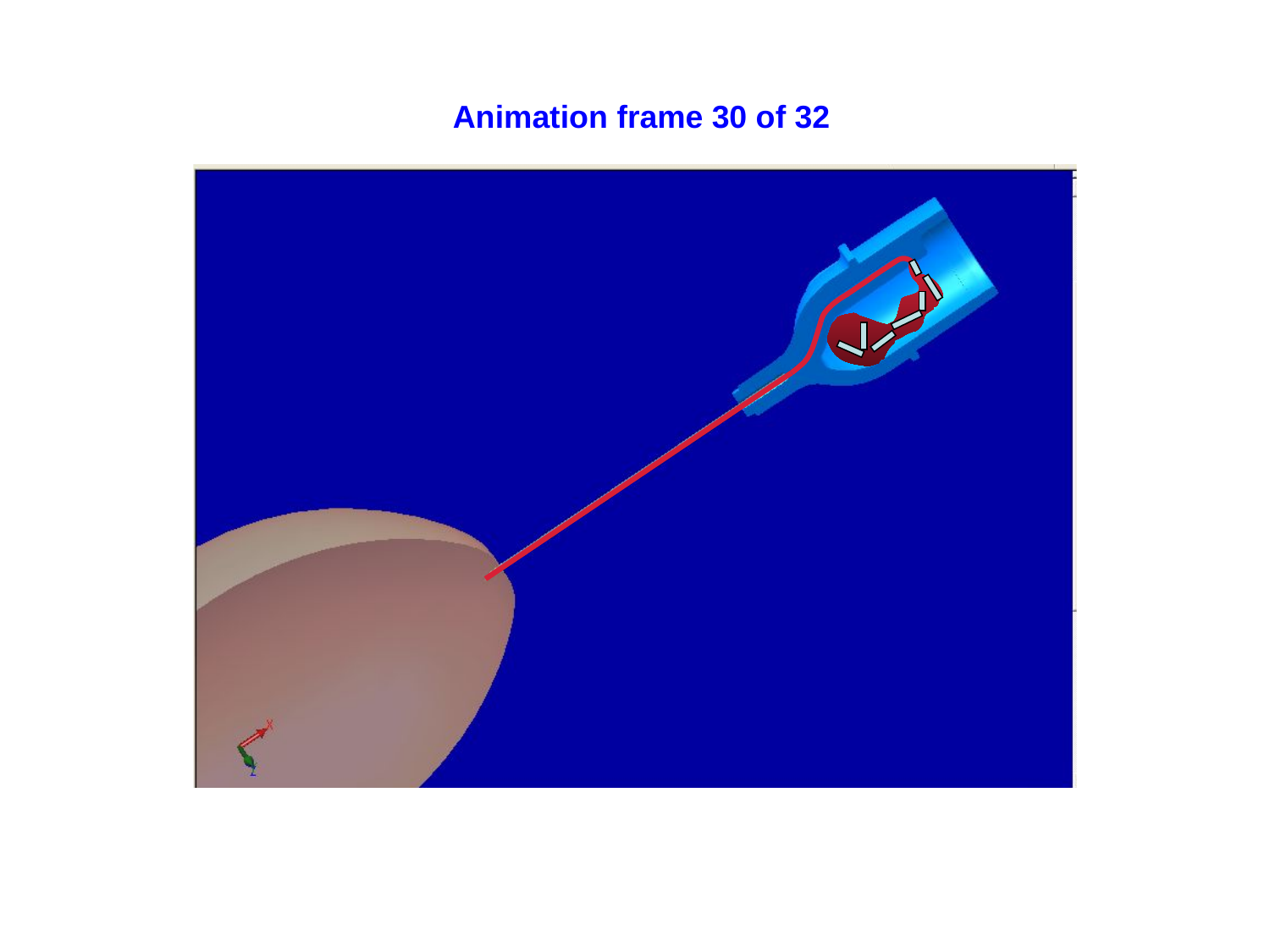

Animation frame 30 of 32

## Slide 31
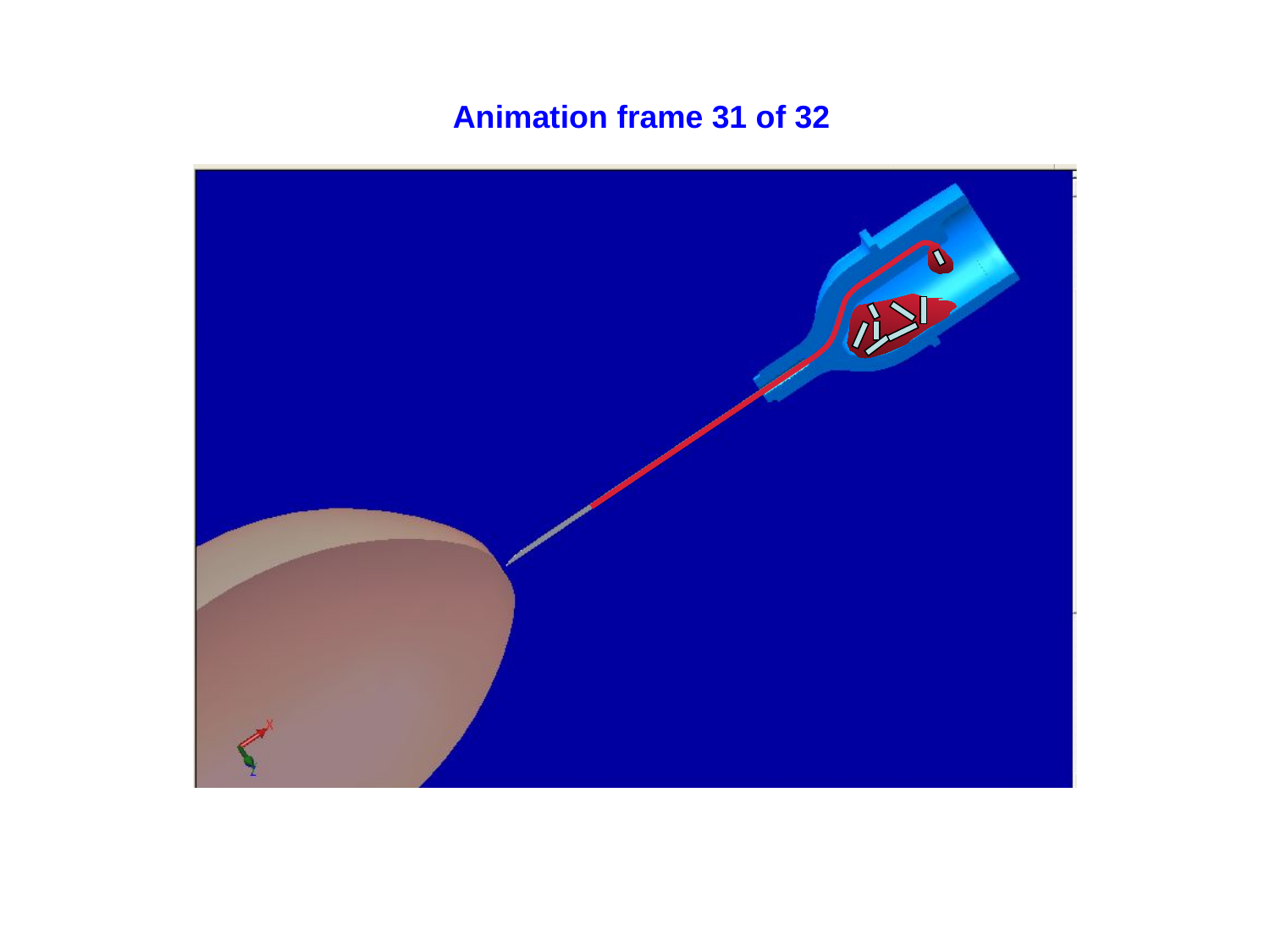

Animation frame 31 of 32

## Slide 32
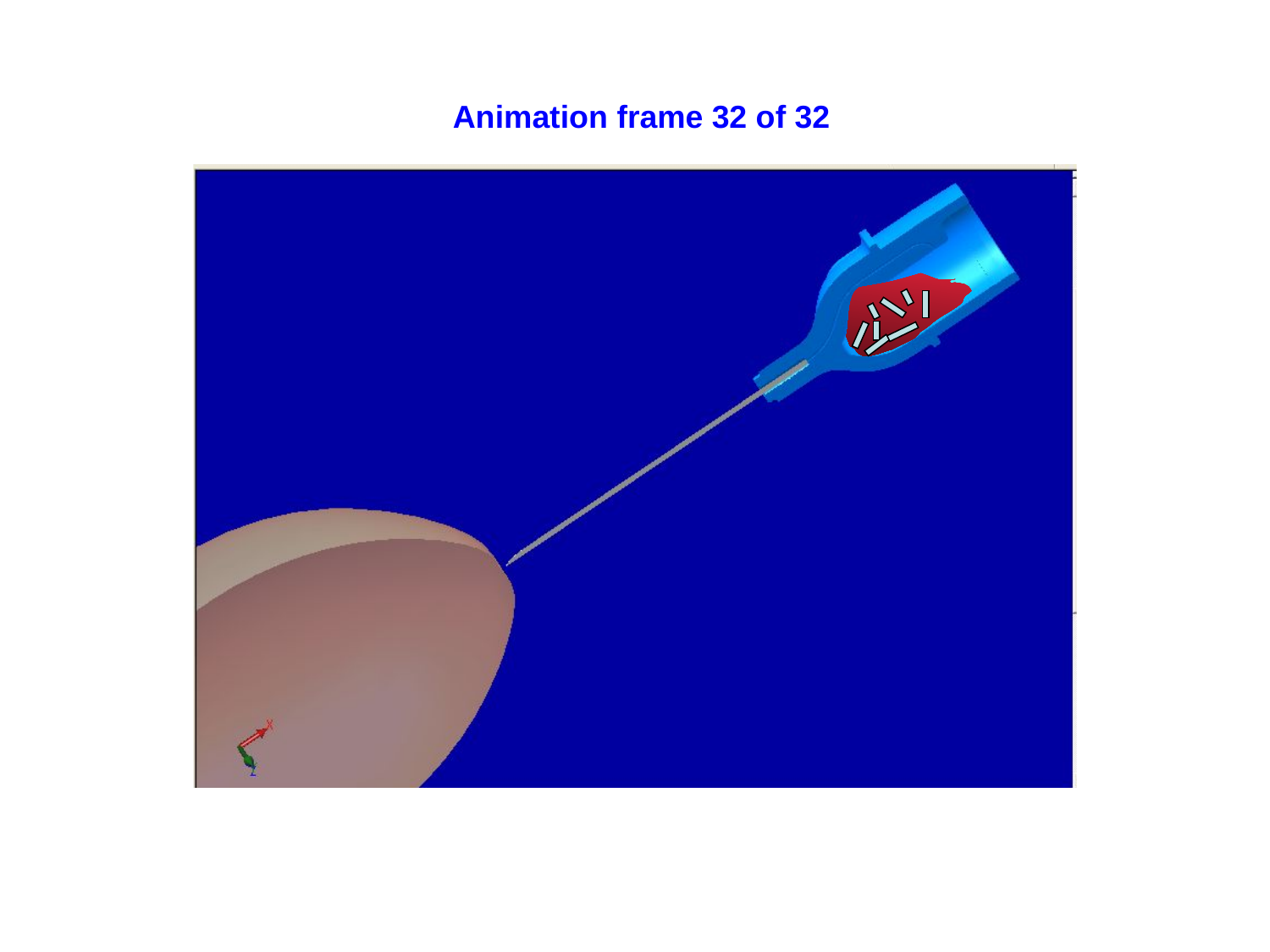

Animation frame 32 of 32
